# Not just like starting over - Leadership and revivification of cooperation in groups

**DOI:** 10.1007/s10683-015-9468-6

**Published:** 2015-09-25

**Authors:** Jordi Brandts, Christina Rott, Carles Solà

**Affiliations:** 1 Instituto de Analisis Economico (CSIC) and Barcelona GSE, Campus UAB, 08193 Bellaterra, Barcelona, Spain; 2 Department of Economics (AE1), School of Business and Economics, Maastricht University, 6200 MD Maastricht, Netherlands; 3 Department of Business, Universitat Autonoma de Barcelona, Campus UAB, 08193 Bellaterra, Barcelona, Spain

**Keywords:** Leadership, Cooperation, Communication, Experiments, C71, C73, C92, D83, J63, L20

## Abstract

We conduct a laboratory experiment to study how, after a history of decay, cooperation in a repeated voluntary contribution game can be revived in an enduring way. Simply starting the repeated game over—a simple fresh start—leads to an initial increase of cooperation, but to a subsequent new decay. Motivated by cooperation decay in organizations we study the potential of three interventions of triggering higher and sustained cooperation, which take place at the same time as a restart. Surprisingly, we find that the detailed explanation of the causes of the decay in cooperation of Fischbacher and Gächter (Am Econ Rev 100:541–556, 2010) combined with an advice on how to prevent decay do not have an effect beyond that of just starting over. In contrast, a one-way free form communication message sent by the leader to the followers strongly revives cooperation. We find evidence that *repeated* free form communication by the leader further strengthens the reviving effect on cooperation. Combining the two previous interventions does not outperform the pure effect of communication. Our content analysis reveals that leader communication is more people oriented than the expert advice.

**Electronic supplementary material:**

The online version of this article (doi:10.1007/s10683-015-9468-6) contains supplementary material, which is available to authorized users.

## Introduction

A common observation in experimental studies of public goods games is that, in environments with a finite horizon, cooperation levels are initially rather high but then decrease steadily over time.^1^ The question we study in this paper is which instruments can be used to revive cooperation effectively after such a history of decay. Salient temporal landmarks, like the start of a new week or a new season, may create a sense of a new beginning in a natural environment and allow for the revivification of cooperation. Dai et al. (2014) discuss in detail how such temporal landmarks can affect individual behavior, like eating more healthy food or saving money. Our focus is on whether such salient temporal landmarks also affect the behavior of groups and whether their effects can be reinforced through some additional interventions. Our motivation for studying these issues comes from the analysis of organizations and the need to find ways to combat organizational decadence.

Our laboratory experiment builds on two important results of earlier experimental work related to the effects of a fresh start in the context of cooperation. First, it has been shown that in fixed groups the level of cooperation can be driven up again by simply restarting the game after the initially announced horizon has been reached. In the experiments reported in Andreoni (1988) participants play the voluntary contribution game in the finitely repeated form. After the initially announced ten rounds are over, they are informed that there will be some additional rounds of the same game. Here the re-initiation of play allows for a fresh start. Contributions go up again after the prolonged experiment is announced. In Andreoni’s (1988) experiment play was suspended after three additional rounds and during these rounds the cooperation level stayed up. This effect is called the “restart effect,” and it is the first of two results on which we build.

The second regularity we build on is reported in Croson (1996) who follows up on Andreoni (1988). In her public goods experiment, ten additional rounds are announced after the initial ten rounds are over. The results confirm that the restart leads to an initial increase of cooperation in fixed groups. However, after the initial increase in cooperation, the decline in cooperation begins again and play ends up at an even lower level than at the end of the first ten rounds. That is, cooperation can be revived by starting over, but the effect is short-lived.

In this paper we use a lab experiment to study how cooperation in groups can be revived *in an enduring way* by using various managerial strategies that come along with a fresh start. As discussed above, a positive reaction to a fresh start seems to be a widespread behavioral regularity. Our focus is on studying whether humans’ spontaneous tendency to react to a fresh start can be reinforced by some additional intervention.

We study this issue in the context of a public goods game involving a leader. We choose such a structure, because we are mostly motivated by issues of successful teamwork in organizations.^2^ Almost all types of institutions, firms, departments and (sport) teams are organized in some kind of hierarchical structure and guided by a leader. When cooperation failure has occurred it is one of leaders’ natural roles to take action to reinforce a new beginning.

In our set-up, leadership takes the form of leading-by-example used in the studies by Güth et al. (2007), Rivas and Sutter (2011), Gächter et al. (2012) and Potters et al. (2007) among others. The question we ask is whether in a leading-by-example environment leaders can take advantage of the tendency of human cooperation to react positively to exogenously set landmarks by taking deliberate action precisely at the natural landmark. Here we study three interventions that can potentially lead to a stronger revivification of cooperation than that following a pure restart and that are interesting from a managerial point of view.^3^

The participants play the public goods game with leading-by-example in fixed groups and repeatedly in 36 rounds. The 36 rounds are divided into three parts with 12 rounds each. In the first part of the experiment we let participants play the game without any intervention. The purpose of the first part is to create the experience of decreasing cooperation in the group and to provide an interesting situation for a restart. The second (third) part serves to measure the short- and long-run effect of a (repeated) restart.

We have four treatments, which all involve a restart in the sense that, after a number of experimental rounds, additional rounds are played. The first is the *pure restart* treatment, a control treatment in which the restart is not accompanied by any other change in the environment and which is meant to establish a baseline. The three remaining treatments involve additional elements that go beyond the pure restart. Our second treatment is the *comprehension*/*advice* treatment, where the restart is combined with the provision of a detailed explanation of the causes of the decrease in cooperation and of advice on how to prevent a decay. Our third treatment is the *communication* treatment, where the restart is accompanied by a one-way free form message sent by the group leader to the followers. In the fourth treatment, the *comprehension*/*advice*/*communication* treatment, we combine the second and third treatment.

Our contribution to the existing literature on cooperation is fourfold: First, we analyze the pure restart in a voluntary contribution game with two new features: the game is sequential and, in contrast to the surprise restart in Andreoni (1988) and Croson (1996), participants in our experiment know that there will be a restart. Second, we study the effect of one-time and one-way communication *after* having possibly experienced cooperation failure, in contrast to repeated communication among all group members in Isaac and Walker (1988) and one-time and one-way communication form the start in Koukoumelis et al. (2012). Third, “expert” advice on how to prevent a decay in cooperation in a voluntary contribution game has to our knowledge not been studied before, though “expert” advice explaining the game theoretic prediction of the voluntary contribution game and the effect of communication has been studied by Brosig et al. (2003). Fourth, the repeated restart allows us to study whether, if the first effect is positive, repeated interventions can further strengthen the initial reaction and lead to sustained cooperation levels.

We find that the effect of communication from the leader to the followers revives cooperation significantly more than the pure restart and the comprehension/advice treatments. There is evidence that the *repeated* communication by the leader (without the expert explanation and advice) further strengthens the positive effect on cooperation. The effect of comprehension and advice is not beyond that of just starting over.

## Experimental design

In Sect. 2.1, we present the sequential voluntary contribution game used in our experiment. In Sect. 2.2 we provide some general information on the procedures of the experimental sessions. In Sect. 2.3, the control treatment and the intervention treatments are discussed.

### The game

In the leading-by-example setting we study, a voluntary contribution game is played repeatedly by fixed groups of four participants. Group members are matched randomly at the beginning of the experiment. There are two roles: one leader and three followers. The role of the leader is randomly assigned to one of the group members at the beginning of the experiment and the remaining group members are followers. Participants keep their role throughout the entire experimental session.

The payoff function is the same for leaders and followers measured in Experimental Currency Units (henceforth, ECU). The individual endowment is *E* = 40, the return rate of the private good is *r*_*P*_ = 1, and the return rate of the public good is *r*_*V*_ = 0.5 yielding the following payoff function of individual *i* in round *t*, where an individual *i*’s contribution in round *t* to the public good is denoted by *h*_*i*,*t*_, the contributions by all group members are denoted by *h*_*j*,*t*_ with *j* = 1,…,4:

$$ \pi_{i,t} = \underbrace {{(40 - h_{i,t} )}}_{\text{Payoff\; from\; private\; good}} + \underbrace {{0.5 \cdot \sum\limits_{j = 1}^{4} {h_{j,t} } }}_{\text{Payoff\;from\; public\; good}} $$

The game has three stages. In the first stage of the game, the leader of each group decides how much of the endowment to contribute to the public good. In the second stage, followers are informed about their leader’s decision and decide each of them independently how much of their individual endowment to contribute to the public good.^4^ In the third stage, all players are informed about the average contribution by the other group members, the sum of contributions by all group members and the individual payoff. The game is played repeatedly in 36 rounds.

### Procedures

At the beginning of an experimental session the general instructions are handed out to the participants on paper and then read aloud by one of the experimenters. In the general instructions (see Online Appendix A.1), the chronological order of an experimental session and the three stages of each round are represented graphically. The general instructions are the same for the control treatment and the three intervention treatments. Before the experiment starts participants are informed that there will be 36 rounds of the voluntary contribution game (divided into three parts with 12 rounds each) and that they will get part-specific instructions at the beginning of each part.

Additional part-specific instructions (see Online Appendices A.2–A.5) are shown on the computer screen just before the corresponding part starts and also announced aloud by one of the experimenters. They include the information that the group composition would remain the same over the 12 rounds of the subsequent part. The restart and the interventions take place at the beginning of part 2 (before round 13) and part 3 (before round 25). A 12-round part can be seen as a work-period (week, month, quarter, year), a season, the time a particular project lasts or any other length of time after which there is a natural break in the interaction.

The experimental sessions were conducted at the Universitat Autònoma de Barcelona (UAB, Spain) and programmed with the experimental software z-Tree, Fischbacher (2007). Participants were mainly undergraduate students from the UAB and were recruited using the online recruitment system ORSEE, Greiner (2004). A total of 208 participants took part in 12 experimental sessions composed by 123 women and 85 men. The conversion rate was 150 ECU to 1 Euro. The average earnings per person were 19.70 Euro (including a show-up fee of 5.00 Euro). The average duration of a session was 2 h 30 min. After the experiment had finished, participants were asked to fill out a questionnaire and were paid their earnings in private.

### Treatments

As mentioned above, the first 12 rounds were identical across treatments. Our conjecture here was that contributions would decrease over time with no difference across treatments.

In the *pure restart* treatment, participants are informed before the start of part 2 and part 3, respectively, that during the subsequent 12 rounds they will continue playing under the same conditions and in the same group composition as before.^5^ They do not get any additional information and do not have to take any new type of action in the second or third part.

In the *comprehension*/*advice* treatment, we add to the information on the fix group composition an explanation and advice text displayed on the computer screens. We explain to participants, before the start of part 2, how contributions usually evolve in related experiments and give an explanation of why they typically decline, following the findings of Fischbacher and Gächter (2010). Then we give advice on what to do to avoid the decline and to reach and maintain high earnings from the public good. The idea behind this treatment is that of a working group receiving external expert analysis, explanation and advice. McDonald and Westphal (2003) for instance find that CEOs tend to seek advice when performance deteriorates, which in our context corresponds to decreasing cooperation. The evidence about the effect of external consultancy and advice on performance is however rather inconclusive as a number of field experiments with micro-, small, and large organizations in developing countries obtain different results.^6^

The content of the explanation given to subjects at the beginning of part 2 is the following: We first tell participants that we observed a decline in average contributions over part 1 in previous sessions driven by followers undercutting previous contributions on average. We then explain to them that a study showed that the decline in contributions in the repeated simultaneous game occurs because participants are on average imperfect conditional contributors (Fischbacher and Gächter 2010). Finally, we state that it is recommendable that followers contribute at least as much as the leader of their group to reach and maintain high group earnings from the public good.^7^ Before part 3, we give a short reminder of the explanation and the recommendation. The full text of the comprehension/advice instructions for part 2 and part 3 can be found in Online Appendix. It was important for us that participants understood the game well and were given a clear, but comprehensive recommendation of how to prevent the decline.^8^ Compared to Brosig et al. (2003) who focus on the game theoretic prediction and the effect of communication in their “lecture” treatment, our comprehension/advice text focuses on the decline of cooperation over time and the sequential game form involving leaders and followers.

The fact that the message is repeated is an important element of our design.^9^ Repetition of the message has been analyzed in the psychological literature which suggests that extended effects on attitude can occur when the initial information on which judgment was based is retrieved (Wood 2000). Moreover message repetition provides more chances to scrutinize the message.

Psychologists have extensively studied attitude change and persuasion (see Petty and Wegener 1998; Wood 2000; Bohner and Dickel 2011). Results of this research suggest that the effect of our comprehension/advice treatment could go both ways. On one hand, the better understanding provided by the message is directed to the desire for accuracy on the object and this could lead to participants changing their attitude and contributing more (Wood 2000). In addition, persuasion effects increase when the message contains strong, cogent arguments (Wood 2000). Finally, the origin of the message is important. Cialdini and Trost (1998) point out that legitimacy provides extreme influential capacity and expertise is one of the ways to acquire legitimacy. Our message stresses that the advice is based on expertise.

However, there are also reasons to think that the treatment will lead to low contributions, since the reaction to the comprehension/advice combination may be defensive. For example, Tycocinski et al. (1994) suggest that certain messages can elicit distress by identifying seemingly relevant goals that have not been adopted. Also, there is the possibility that our message is too complex, and this could weaken its positive effect (Petty and Wegener 1998).^10^

In the *communication* treatment, the leader of a group sends a one-way free form text message to the followers before the start of part 2 and part 3, respectively. Except for standard rules for free form communication in experiments, leaders are free to write whatever they want. We are interested in studying behavior in the sequentially played voluntary contribution game and *after a decrease* in contributions; our emphasis is on reviving cooperation after it has died down. It is an interesting context because after a negative cooperation experience it is particularly crucial that leaders find the right words to get the group out of the trap. Given previous evidence on communication, on could conjecture that communication would increase cooperation by more than the pure restart.^11^ However, some of the caveats presented for the comprehension advice treatment also apply to the communication treatment. In particular, depending on how leaders formulate their messages they can also elicit distress by focusing too much on some negative aspects of followers’ past behavior.

Our communication treatment is related to some previous experimental work. In Isaac and Walker (1988) participants play the simultaneous public good game in two sequences of ten rounds with and without communication. Communication, which takes place among *all* group members and in *each* round, revives cooperation substantially after a sequence without communication. Koukoumelis et al. (2012) have shown that *one*-*way* communication from one group member to the others *from the start* (once before the first round or in each round) increases cooperation significantly. It is an open question whether the same is true for *one*-*way* communication from the leader to the followers taking place *once* (and not in each round) after participants have experienced decreasing cooperation.

In the *comprehension*/*advice*/*communication* treatment, all participants receive exactly the same explanation and advice as in the comprehension/advice treatment before the start of part 2 and part 3, respectively. On the subsequent screen, leaders can then send a one-way free form message to the followers exactly like in the communication treatment. Since this intervention is a combination of the other two our conjecture here is again open; both a positive and a negative effect could emerge.

In the following, we will denote the pure restart control treatment by “treatment PR,” the comprehension/advice intervention by “treatment CA,” the communication intervention by “treatment C,” and the comprehension/advice intervention in combination with the communication by “treatment CAC.” Table 1 provides a summary of the characteristics and the number of groups for each treatment. We have a total of 15 (independent) group observations for treatment PR, 13 group observations for treatment CA, 12 group observations for treatment C, and 12 group observations for treatment CAC.

**Table 1:** Overview over treatments

Treatment	Characteristics	Intervention	Repetitions	Observations
*(Control)* *Treatment PR*	Restart	Before parts 2 and 3	36 rounds	15 groups
*Treatment CA*	Restart and Comprehension and advice text	Before parts 2 and 3	36 rounds	13 groups
*Treatment C*	Restart and One-way free form communication from leader to followers	Before parts 2 and 3	36 rounds	12 groups
*Treatment CAC*	Restart and Comprehension and advice text and subsequently one-way free form communication from leader to followers	Before parts 2 and 3	36 rounds	12 groups

## Results

**Fig. 1:**
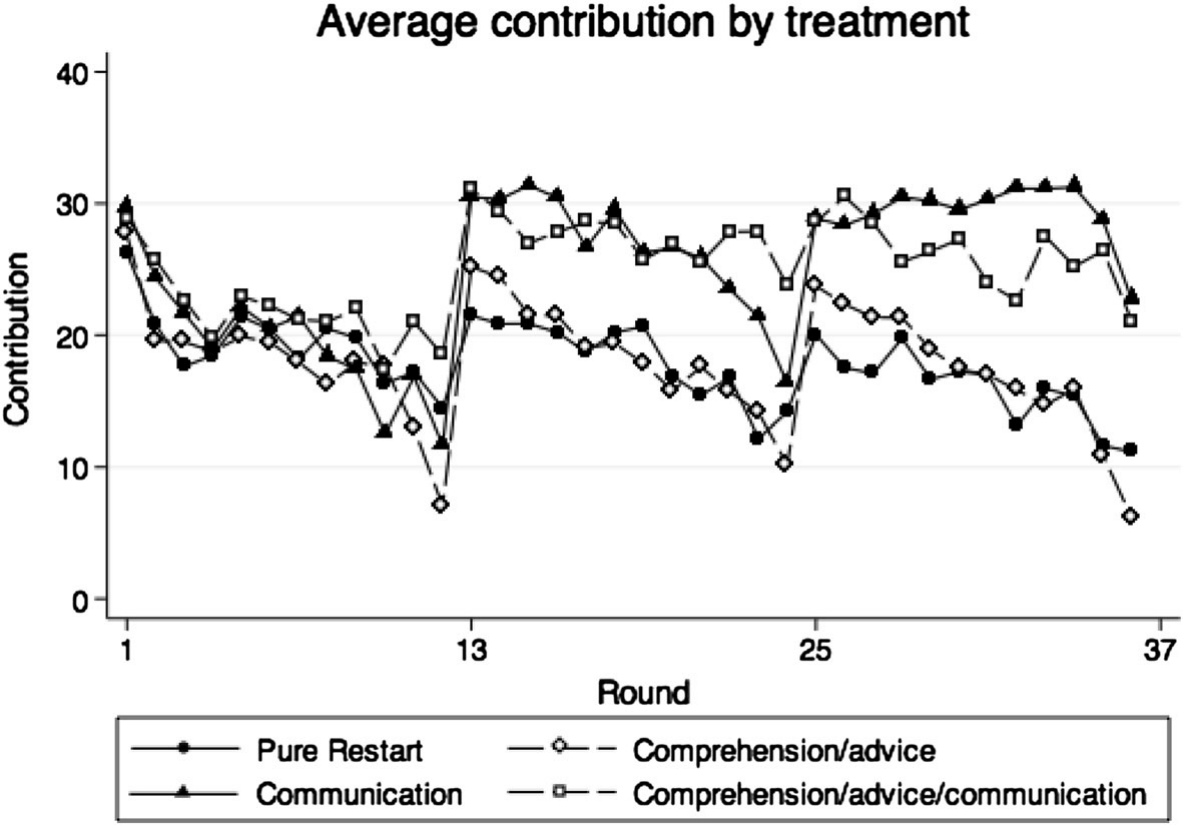
Average contributions in control treatment PR and treatment CA, C, and CAC (round 1–36)

**Fig. 2:**
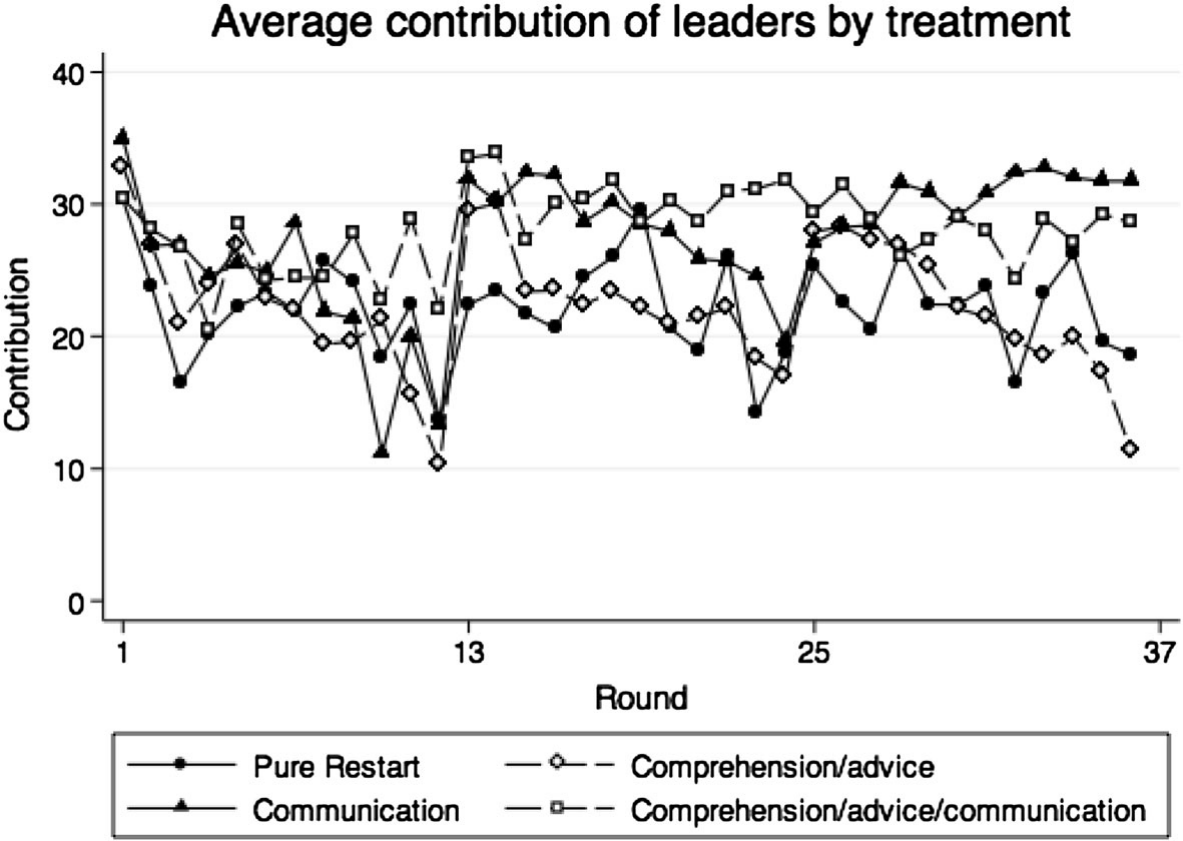
Average contributions of leaders in control treatment PR and treatment CA, C, and CAC (round 1–36)

**Fig. 3:**
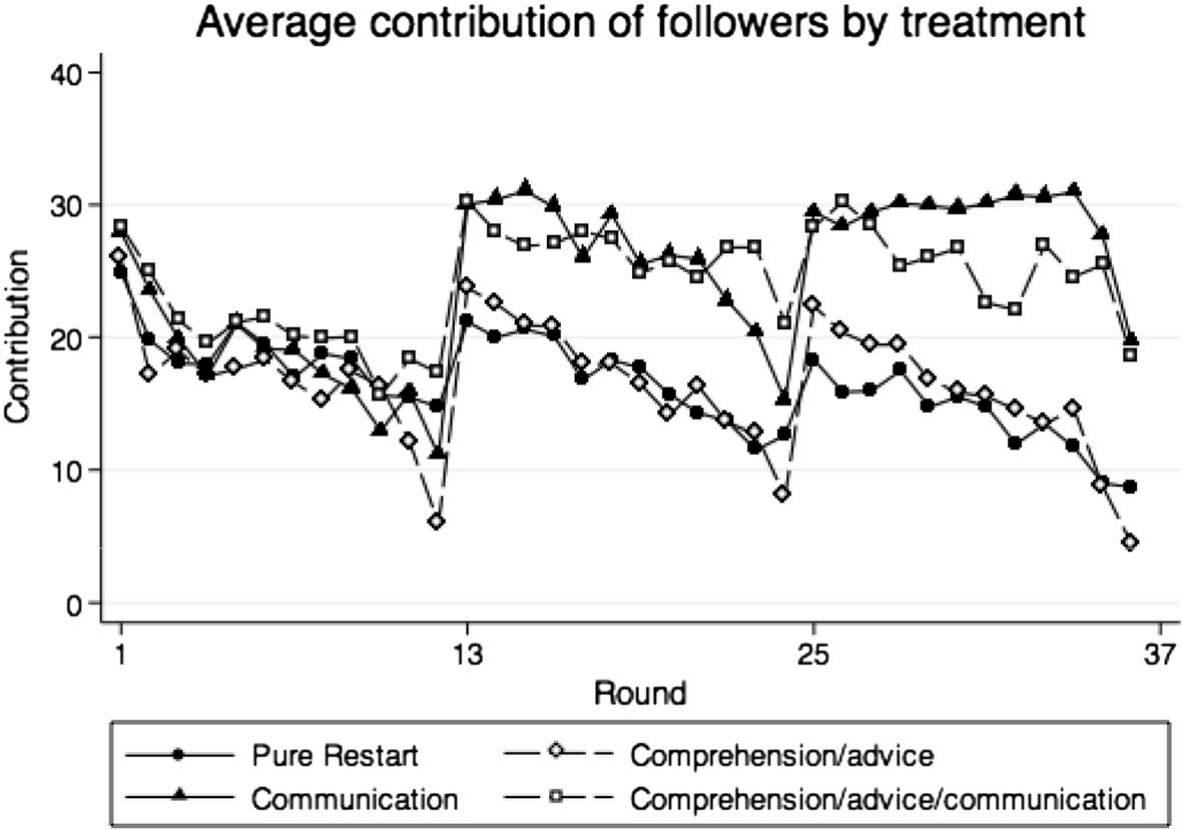
Average contributions of followers in control treatment PR and treatment CA, C, and CAC (round 1–36)

Table 2 shows average contributions and corresponding standard deviations of all participants, leaders and followers in parts 1–3. Figures 1, 2 and 3 show average contributions of group members, average contributions of leaders and average contributions of followers over the 36 rounds of the experiment.

**Table 2:** Descriptive statistics of contributions by treatment and on the group, leader and follower level

Average contributions	N	Group	Leaders	Followers
		Mean (SD)	Mean (SD)	Mean (SD)
Treatment PR				
Part 1 (round 1–12)	15	19.28 (7.442)	21.86 (9.557)	18.41 (7.334)
Part 2 (round 13–24)	15	18.20 (7.588)	22.24 (9.443)	16.85 (7.847)
Part 3 (round 25–36)	15	16.03 (9.769)	22.31 (10.47)	13.94 (10.03)
Treatment CA				
Part 1 (round 1–12)	13	17.86 (7.086)	21.86 (7.321)	16.53 (7.106)
Part 2 (round 13–24)	13	18.51 (9.675)	22.83 (9.919)	17.07 (9.886)
Part 3 (round 25–36)	13	17.12 (10.97)	22.15 (11.78)	15.44 (11.48)
Treatment C				
Part 1 (round 1–12)	12	19.62 (6.068)	23.32 (6.770)	18.39 (6.414)
Part 2 (round 13–24)	12	26.56 (8.364)	28.10 (9.810)	26.04 (8.108)
Part 3 (round 25–36)	12	29.31 (10.32)	30.56 (11.01)	28.89 (10.24)
Treatment CAC				
Part 1 (round 1–12)	12	21.93 (6.714)	25.73 (7.316)	20.67 (7.642)
Part 2 (round 13–24)	12	27.50 (9.725)	30.69 (9.814)	26.44 (10.68)
Part 3 (round 25–36)	12	26.13 (12.16)	28.18 (11.40)	25.44 (12.84)

Group contributions are the average over the contribution of all four member of a group in the 12 corresponding rounds. For leaders, the part contributions are calculated taking the average over the contributions in the 12 rounds of a part on the individual level. For followers, the average part contributions are calculated over the average of the three group followers in the 12 rounds of a part

Table 3 shows the results of pooled OLS regressions. The observations are those of all 208 participants. In all the regressions, we cluster by group to control for the correlation of contributions within a group. In regression models (1a), (1b), (2a), (2b), (3a), and (3b), observations are those from part 1 (rounds 1–12), part 2 (rounds 13–24), and part 3 (rounds 25–36), respectively. In models (1a), (2a), and (3a), individual contributions are regressed on a round variable taking values between 1 and 12 corresponding to parts 1, 2 and 3 respectively, and a dummy variable for each of the three interventional treatments CA, C, and CAC, with PR being the reference treatment. The regressions also include a dummy variable which takes the value one if the individual is a leader and zero if the individual is a follower. In models (1b), (2b), and (3b), an interaction term between the round variable and each of the three treatments CA, C, and CAC is added to the corresponding model. In regression models (4a) and (4b), observations are those from round 1 through 36. In model (4a), the individual contributions are regressed on a round variable taking values between 1 and 36, a dummy variable for part 2 and part 3, respectively, a dummy variable for each of the three interventional treatments CA, C, and CAC, and a dummy variable for the role “leader.” In model (4b), interaction terms between each part dummy and each treatment dummy are added to model (4a).

**Table 3:** Pooled OLS regression (Data: treatments PR, CA, C, and CAC)

Variables	(1a)	(1b)	(2a)	(2b)	(3a)	(3b)	(4a)	(4b)
	Dependent variable: contribution							
	(Part 1)	(Part 1)	(Part 2)	(Part 2)	(Part 3)	(Part 3)	(Part 1–3)	(Part 1–3)
Part round (1–12)	−0.882*** (0.137)	−0.594*** (0.220)	−0.848*** (0.167)	−0.754*** (0.251)	−0.679*** (0.148)	−0.673** (0.261)		
Round (1–36)							−0.803*** (0.101)	−0.803*** (0.101)
Part 2							12.37*** (1.721)	8.557*** (2.018)
Part 3							21.35***	16.03***
							(3.005)	(3.985)
Comprehension/advice	−1.413 (2.676)	1.924 (2.604)	0.311 (3.233)	2.886 (4.385)	1.086 (3.846)	5.288 (5.146)	−0.00513 (2.675)	−1.413 (2.676)
Communication	0.345 (2.529)	4.405 (2.743)	8.358*** (3.020)	10.68** (4.282)	13.28*** (3.793)	9.895** (4.923)	7.328*** (2.390)	0.345 (2.528)
Comp./advice/communication	2.658 (2.653)	3.107 (2.858)	9.305*** (3.323)	6.849* (3.827)	10.10** (4.197)	9.089* (4.807)	7.353** (2.910)	2.658 (2.653)
(CA)*(Part round)		−0.513 (0.335)		−0.396 (0.380)		−0.646* (0.368)		
(C)*(Part round)		−0.625** (0.299)		−0.357 (0.534)		0.521* (0.308)		
(CAC)*(Part round)		−0.0691 (0.426)		0.378 (0.368)		0.155 (0.449)		
(CA)*(Part 2)								1.723 (2.801)
(CA)*(Part 3)								2.499 (4.196)
(C)*(Part 2)								8.013** (3.007)
(C)*(Part 3)								12.94*** (4.382)
(CAC)*(Part 2)								6.647** (2.741)
(CAC)*(Part 3)								7.440* (3.936)
Leader	4.631*** (0.804)	4.631*** (0.805)	4.453*** (0.928)	4.453*** (0.929)	5.107*** (0.960)	5.107*** (0.961)	4.730*** (0.768)	4.730*** (0.768)
Constant	23.85*** (1.952)	21.98*** (2.047)	22.60*** (2.336)	21.99*** (2.929)	19.17*** (2.809)	19.13*** (3.507)	20.27*** (1.932)	23.31*** (1.935)
Observations	2496	2496	2496	2496	2496	2496	7488	7488
R-squared	0.073	0.077	0.129	0.134	0.157	0.165	0.110	0.128

Robust standard errors in parentheses

Pooled OLS (clustering for group), observations from rounds 1-12 (regression models 1a and 1b), rounds 13-24 (regression models 2a and 2b), rounds 25-36 (regression models 3a and 3b), rounds 1-36 (regression models 4a and 4b)

Dependent variable (contribution) takes values between 0 and 40

*** p < 0.01; ** p < 0.05; * p < 0.1

Sections 3.1, 3.2, and 3.3 deal with the contribution levels of parts 1, 2 and 3 respectively. We focus on contributions of complete groups and where appropriate distinguish between leaders and followers. Throughout the paper, we use (average) contributions on the group level as independent observations for the non-parametric tests. In Sect. 3.4 we study the content of leader communication.

### Part 1 (rounds 1–12)

Consider the information pertaining to part 1 in Table 2. Using average contributions on the group level as independent observations, we find that, as expected, the null hypothesis of no treatment differences in contributions in part 1 cannot be rejected (p = 0.592, Kruskal–Wallis test). Also the pair-wise comparisons of part 1 contribution distributions do not reveal differences between treatments PR, CA, C, and CAC (p > 0.210, pair-wise Mann–Whitney U test).^12^

Contributions in part 1 are also the same across treatments when analyzing leaders (p = 0.708, Kruskal–Wallis test; p > 0.255, pair-wise Mann–Whitney U test) and followers (p = 0.573, Kruskal–Wallis test; p > 0.191, pair-wise Mann–Whitney U test) separately. The absence of treatment differences is confirmed in regression model (1a), where the coefficient estimates of the treatment dummy variables CA, C, and CAC are all not significant at conventional levels.

Figures 1, 2 and 3 show that cooperation declines in part 1 (rounds 1–12) and this is confirmed in regression models (1a) and (1b) in Table 3. The results for model (1a) in Table 3 show that the coefficient estimate for the round variable is negative and highly significant at the one percent level indicating that contributions decrease over the rounds of part 1 by 0.88 ECU per round on average. In model (1b) the dummy variables for the three treatments are again not significant at conventional levels. All three interaction terms of the treatment and the round variable are negative. For treatment C, the interaction term is significant at the five percent level in part 1. Compared to control treatment PR, contributions start somewhat higher in treatment C in round 1 and the contribution decrease in part 1 is steeper by 0.63 ECU per round.^13^

Comparing leaders’ and followers’ contributions with the data of Table 2, we find that leaders contribute in part 1 significantly more than the followers of the corresponding group in each treatment (p < 0.084 for each treatment separately, Wilcoxon signed-rank tests). The larger contributions of leaders in part 1 are confirmed in regression models (1a) and (1b) in Table 3. This replicates an earlier finding by Güth et al. (2007), Potters et al. (2007) and Gächter et al. (2012). In our data, leaders contribute on average 4.6 ECU more than followers.

Summarizing, in part 1 there are no treatment differences in contribution levels, which decline over the range of the 12 rounds. This result sets the stage for our analysis of the effects of the restarts in the different treatments.

### Part 2 (rounds 13–24)

#### The short-run effect of the first restart

The increase in group contributions from round 12 to round 13 is on average (with the corresponding standard deviation) 7.02 ECU (10.8), 18.13 ECU (9.8), 18.81 ECU (15.0), and 12.60 ECU (11.1) in treatments PR, CA, C, and CAC, respectively. The significance of these increases is confirmed by non-parametric tests (p < 0.061 separately for each treatment and for average group contributions, leaders’ contributions, and average followers’ contributions, Wilcoxon signed-rank test).^14^

Next we ask whether there are treatment differences in the first restart effect, i.e. in the contribution *increase*. The differences between the increases in contributions can be observed in Figs. 1, 2 and 3. The increase in treatments CA and C is significantly larger than the increase in the control treatment PR (p = 0.015 and p = 0.038, respectively, Mann–Whitney U test), while the difference is not significant for treatment CAC compared to PR.^15^

For treatment CA, the increase is significantly higher than in control treatment PR also for leaders and followers separately. For leaders the comparison between contribution increase in treatments CA and PR is 19.15 ECU vs. 8.73 ECU, (p = 0.074, Mann–Whitney U test); for followers, the comparison is 17.79 ECU vs. 6.44 ECU (p = 0.020, Mann–Whitney U test). For treatment C, the contribution increase is significant for followers (18.92 ECU vs. 6.44 ECU, p = 0.043, Mann–Whitney U test), but not for leaders.

What happens in the long-run in part 2, i.e. in rounds 13 through 24? In what follows we present two distinct comparisons. First, we compare the *contribution levels* in part 2 across treatments. Second, we look at differences-in-differences and see whether the *changes* in contribution levels between complete parts 1 and 2 are different across treatments.

#### Average contributions over all rounds of part 2 across treatments

Overall, contributions decline over the 12 rounds of part 2 in all four treatments, see regression models (2a) and (2b) in Table 3. The effect of the first restart is short-lived in all treatments.^16^ The next question is whether average contributions over all rounds of part 2 are higher in some treatments than in others.

We find that contributions in part 2 (rounds 13–24) are highest when the leader communicates with the followers (irrespective of the additional comprehension/advice text), whereas they are similar in the pure restart and the comprehension/advice intervention, see Fig. 1 and Table 2. Contributions in either treatment with communication are significantly higher than in treatments PR and CA (p < 0.045, pair-wise Mann–Whitney U test), whereas there are no significant differences in the distribution of average group contributions between control treatment PR (18.20 ECU) and treatment CA (18.51 ECU) (p = 0.695, Mann–Whitney U test), or between treatment C (26.56 ECU) and treatment CAC (27.50 ECU) (p = 0.773, Mann–Whitney U test). That is, in the short-run the contribution increase in CA is large, but this does not prevent the long-run contribution level over all 12 rounds of part 2 to be lower in CA than in both C and CAC.

Separate analyses for leaders and followers draw a similar picture; see also Figs. 2 and 3 and Table 2. For leaders, contributions in treatment CAC are significantly larger than in treatment PR and CA (0.014 < p < 0.041, pair-wise Mann–Whitney U test) suggesting that leaders try to push contributions up in treatment CAC. Leader contributions in treatment C in part 2 are somewhat larger than in treatment PR and CA, but not significantly (0.143 < p < 0.211, pair-wise Mann–Whitney U test). Followers contribute significantly more in the communication treatments C and CAC than in treatments PR and CA (p < 0.039; for the four pair-wise Mann–Whitney U tests). There are no differences between control treatment PR and treatment CA as well as treatment C and treatment CAC for leaders only (p = 0.982 and p = 0.339, pair-wise Mann–Whitney U test) and for followers only (p = 0.730 and p = 0.730, pair-wise Mann–Whitney U test).

The regression models (2a), (2b), (4a) and (4b) in Table 3 confirm the effect of communication beyond the pure restart effect.^17^ The dummy variables for treatments C and CAC are significant at the ten to one percent level and show that contributions in the communication treatments in part 2 are on average 8 ECU (treatment C) and 7–9 ECU (treatment CAC) larger than in the control treatment with pure restart, see models (2a) and (4b). The coefficient estimates of the dummy variable for the other intervention treatment CA are insignificant.^18^ Note that, in model (2b), the coefficient estimates of the three interaction terms are insignificant; in part 2 cooperation declines over time similarly in all treatments.

Summarizing, communication by the leader does not prevent a decline of contributions over time, which also occurs in the pure restart and the external comprehension/advice interventions (see regression model 2b), but leads to an overall higher level of cooperation in part 2.^19^

#### Changes between parts 1 and 2 across treatments

From part 1 to part 2, average group contributions increase in treatment C (+6.94 ECU) and treatment CAC (+5.57 ECU), remain almost the same in treatment CA (+0.65 ECU), and decrease slightly in treatment PR (−1.08 ECU).^20^ The rise in cooperation from part 1 to part 2 is significantly larger in treatments C and CAC than in control treatment PR (p = 0.032 and p = 0.017, respectively, Mann–Whitney U test), but not significantly larger than in treatment CA (p = 0.135 and p = 0.115, respectively, Mann–Whitney U test).^21^ Cooperation changes in treatments CA and PR do not differ (p = 0.596, Mann–Whitney U test).

Looking at leaders only, there are no significant differences in the contribution reaction to any of the three interventions or to the pure restart (p > 0.107, pair-wise Mann–Whitney U test). The change in cooperation is significantly larger among followers in treatment C compared to treatments PR and CA (p = 0.015 and p = 0.082, respectively; Mann–Whitney U test). Adding communication (treatment CAC) to the comprehension/advice text (treatment CA) does not increase the followers’ contribution significantly (p = 0.157, Mann–Whitney U test) nor does adding the comprehension/advice text (treatment CAC) to the communication (treatment C) that followers receive from the leader (p = 0.954, Mann–Whitney U test). These findings indicate that the effects of communication and comprehension/advice do not have an additive effect. Adding the comprehension/advice text to the communication opportunity seems to rather weaken the positive effect of communication on followers’ contributions. There are no significant differences between treatment PR and CA among followers (p = 0.461, pair-wise Mann–Whitney U test).

In summary, comparing part 2 with part 1 as a whole we find that communication by the leader leads to a strong increase in cooperation in treatments C and CAC, i.e. communication is effective independently of the expert explanation and advice. The increase in contributions with the expert explanation and advice in treatment CA does not differ from the one in treatment PR.

### Part 3 (rounds 25–36)

#### The short-run effect of the second restart

The increase in group contributions from round 24 to round 25 is on average (with the corresponding standard deviation) 5.87 ECU (12.9), 13.54 ECU (12.3), 12.5 ECU (16.9), and 4.8 ECU (7.9) in treatments PR, CA, C, and CAC, respectively (see also Figs. 1, 2 and 3). Note that the contribution levels in round 24 vary considerably across treatments, which is because the first treatment intervention takes place between rounds 12 and 13. Therefore the treatment effects at the second restart cannot be interpreted as pure treatment effects on cooperation (like at the first restart), but rather as the enduring or reinforcing effect of the *repeated* treatment interventions. In the control treatment PR, the increase is not significant (p > 0.132 separately for average group, leaders’, and followers’ contributions, Wilcoxon signed-rank test). In contrast, the augmentation is significant for the three intervention treatments CA, C, and CAC (p < 0.084 separately for each intervention treatment and for group, leaders’, and followers’ contributions, Wilcoxon signed-rank test), except for leaders in treatment CAC (p = 0.652, Wilcoxon signed-rank test).

Comparing increases across treatments, the *increase* is significantly larger in treatment CA than in the control treatment PR (p = 0.065, Mann–Whitney U test), but not for treatments C and CAC compared to treatment PR (p > 0.231, Mann–Whitney U test). At the second restart, the comprehension/advice intervention leads to a new short-run reviving effect, while communication does not boost cooperation significantly, in contrast to what happened at the first restart. This is the only comparison, where we do not find superiority of communication over comprehension advice.

There are some differences for leaders and followers separately. For leaders, the short-run *change* is significantly smaller in treatment CAC than in treatments CA and C (p < 0.081, Mann–Whitney U test), which is due to the fact that contributions in treatment CAC decreased slightly less over part 2. Among followers, contributions in treatment CA (14.36 ECU) rise more than in control treatment PR (5.67 ECU) (p = 0.029, Mann–Whitney U test).

#### Average contributions over all rounds of part 3 across treatments

Figure 1 shows that in part 3 average contributions are highest in treatment C, somewhat lower in CAC and lowest in both PR and CA. This impression is largely confirmed by our statistical tests. In treatment C, contributions in part 3 are significantly larger than in control treatment PR and treatment CA (p < 0.009; pair-wise Mann–Whitney U test). The differences between treatment CAC and treatments PR and CA are also positive, but not (as) significant though (p = 0.107 and p = 0.064, respectively; pair-wise Mann–Whitney U test). There are no significant differences in contributions between the control treatment PR (16.03 ECU) and treatment CA (17.12 ECU) nor are there differences between treatment C (29.31 ECU) and CAC (26.13 ECU) (p = 0.908 and p = 0.453, pair-wise Mann–Whitney U test). The regression models (3a) and (4b) in Table 3 confirm the results of the non-parametrics. In model (3a) the coefficients for C and CAC are highly significant and so are the coefficients for the interaction terms between part 3 and both C and CAC in (4b).^22^

Separate analyses for leaders and followers draw a similar picture, see also Figs. 2 and 3 and Table 2. Contributions of leaders (p < 0.074, pair-wise Mann–Whitney U test) and followers (p < 0.005, pair-wise Mann–Whitney U test) are significantly higher in treatment C than in treatments PR and CA. The leaders’ and the followers’ contributions in treatment CAC move somewhere in between the contributions in treatments PR and CA (p < 0.200, pair-wise Mann–Whitney U test) and treatment C (p > 0.462, pair-wise Mann–Whitney U test). There are no significant contribution differences in part 3 between the control treatment PR and the treatment CA for leaders (p = 0.963, Mann–Whitney U test) and for followers (p = 0.982, Mann–Whitney U test).

The regressions also show that in the communication treatment C there is no decay in part 3. In model (3b) in Table 3 the coefficient estimate of the interaction term of the treatment C dummy and the part round variable is positive and significant at the ten percent level. Repeated communication prevents the decrease in contributions over time in part 3 to a large extent: in model (3b), the coefficient estimates of the part round variable and of the interaction term are −0.673 and +0.521, respectively.^23^ In contrast, the coefficient capturing the interaction of round and CAC is not significant and the one corresponding to CA is significantly negative at the 10 % level.

Fixed effects regressions to control for group effects (robust standard errors) of individual contributions on the round variable for each part and each treatment separately confirm that part 3 in treatment C is the only case where the contribution decay over rounds is not significantly different from zero (see Online Appendix A.6 for the regression results). For all other cases, the decay is significantly different from zero on the 1 % level (treatments R and CA separately for parts 1–3; treatment C for parts 1 and 2; treatment CAC for part 1) and on the 5 % level (treatment CAC for parts 2 and 3, p = 0.051 and p = 0.047, respectively). Isaac and Walker (1988) find the same pattern though their experimental setup is somewhat different (simultaneous game form, communication by all group members in every round): after failure of cooperation without communication, contributions to the public good increase with repeated communication.

The positive effect of communication in treatment C on contributions is to a large extent related to the following behavior of followers with respect to the group leader’s contribution. The long-run cooperation reaction in treatment C is particularly strong among followers. Leaders’ contributions are in general significantly larger than followers’ contributions except for treatment C in part 2 (p = 0.170, Wilcoxon signed-rank test). The average contribution gap is cut to more than half from 4.93 ECU in part 1 to 2.06 ECU in part 2 and 1.67 ECU in part 3 (Table 2) meaning that, in treatment C, leaders manage to bring followers’ contributions closer to that of leaders. For treatment CAC, the decline in the average contribution gap is much weaker (5.06 ECU in part 1, 4.25 ECU in part 2, and 3.74 ECU in part 3), but becomes insignificant in part 3 (p = 0.182, Wilcoxon signed-rank test).

Summarizing, like in part 2 average contributions in part 3 are highest if the leader sends a communication message to the followers, whereas they are very similar with the pure restart and the comprehension/advice intervention. In addition, there is no decay over rounds in treatment C except in the last two rounds, due to the well-known end effect.

#### Changes between parts 2 and 3 across treatments

The change in average contributions from part 2 to part 3 is negative in treatment PR (−2.17 ECU), in treatment CA (−1.39 ECU), and treatment CAC (−1.37 ECU) and positive in treatment C (+2.75 ECU). We interpret the treatments effects at the second restart as the enduring or reinforcing effect of the *repeated* interventions rather than as pure treatment effects on cooperation (as in the case of the first restart). Comparing these changes across treatments (differences-in-differences analysis), we find significant differences only for treatment C compared to treatment PR (p = 0.083, Mann–Whitney U test). Leaders who communicate with the followers (treatment C) contribute more than leaders in treatments CA, but not quite significantly so (p = 0.103, Mann–Whitney U test). Followers react significantly more positively to the text message by the leader (treatment C) than to the pure restart (p = 0.054, Mann–Whitney U test).

The lasting effect on cooperation of the leaders’ (repeated) communication with the followers is also confirmed in regression models (4a) and (4b) where the coefficient estimates of the communication dummy (treatment C) and of the interaction term between the treatment C and the part 3 dummies are significant at the one percent level, respectively. The repetition of communication in part 3 does not only maintain the previous reviving effect of the text message, but reinforces it: compared to the pure restart, contributions in treatment C are on average 8 ECU higher in part 2, model (2a), and 13 ECU higher in part 3, model (3a) in Table 3.^24^ The combination of “expert” explanation and advice and leader communication also increases cooperation compared to PR, but does not perform as well as communication by itself.

Summarizing part 3, we find that *repeated* communication (without the explanation and advice stage) *reinforces* the reviving effect of communication on cooperation. It is the only intervention that exhibits an increase of cooperation in part 3 compared to part 2. In the remaining treatments PR, CA, and CAC, average contributions do not change compared to part 2 and decrease over rounds.

### Observations about the communication content

Since the communication is free-form, we can study what kinds of messages the leaders send and whether they differ between treatment C and treatment CAC. We therefore coded the text messages sent in rounds 13 and 25 to their followers. Table 4 summarizes the information about communication separately for rounds 13 and 25 and treatments C and CAC, respectively. The first two rows refer to the time in seconds that leaders need until they enter the last part of their text message and to the average number of words per text message.

**Table 4:** Average of coded values for each summary statistic and communication category in treatments C and CAC in rounds 13 and 25

	Round 13		Round 25	
	Treatment C	Treatment CAC	Treatment C	Treatment CAC
Summary statistics				
Time for message (in s)	303.1	264.2	220.8	192.8
Number of words	72.6	55.1	79.4	72.5
Content of comprehension/advice message				
Observation of decline (0 = no, 1 = yes)	**25.0** **%**	**9.1** **%**	41.7 %	25.0 %
Observation of followers undercutting (0 = no, 1 = yes)	25.0 %	18.2 %	41.7 %	50.0 %
Undercutting reasons (e.g. selfishness) (0 = no, 1 = yes)	16.7 %	18.2 %	**50.0** **%**	**8.3** **%**
Consequences (Future repercussions of actions) (0 = no, 1 = yes)	16.7 %	18.2 %	33.3 %	33.3 %
Conformity (0 = no, 1 = yes)	58.3 %	72.7 %	66.7 %	75.0 %
Payoff-related arguments				
Suggestion (0 = no, 1 = yes)	83.3 %	90.9 %	66.7 %	83.3 %
Efficient suggestion (0 = no, 1 = yes)	41.7 %	36.4 %	33.3 %	33.3 %
Payoff calculation (0 = no, 1 = yes)	41.7 %	36.4 %	25.0 %	41.7 %
Group payoff maximization (0 = no, 1 = yes)	66.7 %	72.7 %	50.0 %	66.7 %
Satisfaction (e.g. benefit for each) (0 = no, 1 = yes)	75.0 %	81.8 %	66.7 %	66.7 %
Strategy (the entries present the number of times a strategy was mentioned for each treatment)	**3 tit-for-tat, 2 grim trigger, 1 random**	**2 tit-for-tat**	1 tit-for-tat, 1 two-tit-for-tat, 2 grim trigger	2 tit-for-tat, 1 grim trigger, 1 random
Social preference, emotions, and willingness to contribute				
Fairness (0 = no, 1 = yes)	**50.0** **%**	**18.2** **%**	**58.3** **%**	**25.0** **%**
Team spirit (0 = no, 1 = yes)	33.3 %	27.3 %	**25.0** **%**	**50.0** **%**
Notification of low contributors (0 = no, 1 = yes)	**33.3** **%**	**18.2** **%**	50.0 %	58.3 %
Praise (0 = no, 1 = yes)	0.0 %	0.0 %	**50.0** **%**	**16.7** **%**
Complaint (0 = no, 1 = yes)	**25.0** **%**	**9.1** **%**	33.3 %	25.0 %
Mood (−1 = bad, 0 = neutral, 1 = good)	**0.33**	**0.18**	**0.33**	**0.17**
Leave contribution decision to followers (0 = no, 1 = yes)	**16.7** **%**	**0.0** **%**	**25.0** **%**	**0.0** **%**
Promise (0 = no, 1 = yes)	25.0 %	18.2 %	25.0 %	16.7 %
Willingness to contribute more than followers (0 = no, 1 = yes)	**25.0** **%**	**0.0** **%**	**16.7** **%**	**0.0** **%**
Other				
Labor notion (0 = no, 1 = yes)	33.3 %	36.4 %	**16.7** **%**	**41.7** **%**
Strange/nonsense (0 = no, 1 = yes)	**25.0** **%**	**9.1** **%**	**8.3** **%**	**25.0** **%**

The number of analyzed text messages in round 13 in treatment CAC is 11 (due to technical problems, the message of one leader was not saved). In all other cases, 12 text messages were analyzed, respectively. The bold value pairs show a considerable difference in communication between the two treatments (ca. 50 % or more)

For the communication content analysis, we mostly adopted the coding categories from Koukoumelis et al. (2012) and added some categories that we thought would be important for our design.^25^ The main code groups are described below. For a more detailed explanation of the coding categories, see Online Appendix A.7.

The first five coding categories in Table 4 refer to communication content related to that of the (pre-determined) comprehension/advice message in treatment C (and CAC). The next six categories involve payoff-related arguments. The third group of coding categories encompasses social preferences, emotional expression, and own contribution behavior. The last group includes communication content that is not related to the experiment and the use of labor notions in the text messages.^26^ The number of analyzed text messages in round 13 in treatment CAC is 11, in all other cases 12 text messages were analyzed.^27^

In treatment C the comprehension/advice categories are mentioned more frequently in round 25 than in treatment 13. Whereas in round 13 each of the five categories is mentioned in between 17 and 58 % of the cases, the respective frequencies go up from 33 to 67 % of the cases in round 25. Some of the communication content in treatment C is thus similar to the content of the expert explanation and recommendation in treatment CAC.

Note that in the comprehension/advice communication text, we only recommend conformity to reach high earnings, but no particular contribution level. Under payoff-related argument one can see that both in treatments C and CAC, 83–91 % (67–83 %) of the leaders make a contribution suggestion in round 13 (25). However, the suggestion to contribute the full endowment is much less frequent in round 13, with only 36–42 % of the leaders suggesting that everybody contributes the entire endowment. The monetary benefit of cooperating is however stressed by almost all leaders (group payoff maximization and satisfaction).

For the “strategy” category we find some suggestive differences between treatment C and treatment CAC. In treatment C, leaders propose more often less forgiving strategies, in particular when communicating for the first time in round 13. Three leaders announce the tit-for-tat, two leaders the grim trigger, and one leader the random strategy in treatment C compared to two tit-for-tat announcements in treatment CAC in round 13. The gap becomes closer in round 25 (one tit-for-tat, one two-tit-for-tat, two grim trigger in treatment C; two tit-for-tat, one grim trigger and one random strategy in treatment CAC).

Moving to the last set of categories one can see that the reference to fairness and the expression of emotions in form of complaint or praise is more frequent without the expert analysis and advice. With communication only, leaders refer more often to fairness reasons (50 and 58 % of the cases in rounds 13 and 25, respectively) compared to treatment CAC (18 % in round 13 and 25 % in round 25). The differences in announced strategies and fairness considerations lead us to conjecture that leaders are more pro-active in their communication in treatment C than in treatment CAC. It is notable that half of the leaders in treatment C praise the observed contributions in round 25 whereas only 17 % of the leaders in treatment CAC do so even though the contributions in part 2 are similar in both treatments. This might be the result of the leader’s cooperation expectations in treatment C being positively confirmed or outperformed or leaders feeling more responsible for the motivation in treatment C. Even though, in round 13, leaders complain more often about the followers previous contributions in treatment C (25 % compared to 9 % in treatment CAC), the overall mood in the text messages is more positive in both rounds.

While none of the leaders in treatment CAC leave the contribution choice explicitly to the followers, some leaders do so in treatment C (17 % in round 13 and 25 % in round 25) or express the willingness to contribute more than the followers (25 % in round 13 and 17 % in round 25) when they are not influenced by the expert analysis and advice. Leaders express clearly more emotional closeness and voluntariness in treatment C than in treatment CAC.

Summarizing the communication content, we find that the expression of emotions in form of complaints or praise is more frequent in treatment C than in treatment CAC. In particular before the second restart, leaders praise more often the observed contributions. They also leave more autonomy to followers and stress more often fairness considerations. The comprehension/advice categories are mentioned frequently in treatment C, in particular when leaders communicate a second time. The announced punishment strategies are stronger in treatment C than in treatment CAC.

## Conclusion

Our results show that leader communication with the followers is by far the most effective intervention for increasing cooperation in the long-run. The effect on cooperation is significantly larger than the effect of a pure restart driven mainly by increased contribution of followers. The effect is also larger compared to an external expert explanation and advice based on the study by Fischbacher and Gächter (2010).

A combination of the expert explanation and advice together with the leaders’ communication with the followers increases cooperation, but does not outperform the pure effect of communication on cooperation. In addition, *repeated* communication (without the expert explanation and advice) *reinforces* the reviving effect of communication on cooperation. After the leader sends a second text message to the followers, contributions increase immediately and barely decay over time. Repeated communication after the comprehension/advice intervention does not have a similar reinforcing effect, but maintains high contribution levels.

The expert consultancy does not show an effect that goes significantly beyond that of a restart in our experiment nor does it improve the effect of the leader’s communication with the followers. What our results show is that the effect is short-lived and that even the short-run effect does not go beyond that of a pure restart. We believe that these negative results are as important as the positive one mentioned above. It is perhaps most surprising that the comprehension/advice treatment has no additional effect, since it would seem that an analysis of the causes of cooperation decline and a clearly formulated advice are the best starting point for not running into the same problem as before. However, as discussed in Sect. 2.3, the information provided in the comprehension/advice may cause distress and trigger a defensive reaction.

One explanation for our finding may be that what matters for cooperation is not *production oriented communication*, as contained in the comprehension/advice intervention and mostly in the communication following the expert explanation and advice, but *people oriented communication* as in the communication only intervention. In a similar vein, one could think about the formal, production oriented expert analysis and advice from an external human resource consulting firm as a way to create a short-run restart in the firm. Whether the external expert advice has an effect beyond the restart may depend on the content of the analysis, the advice, and the communication form.

As to the content of the communication from leader to followers, we do not have enough observations to do a thorough analysis (nor is it the purpose in this study). However, the most commonly mentioned categories are the monetary benefit from cooperation and requesting conditional contribution. Some leaders also threaten to decrease their contribution if the followers do not cooperate at the same level, create a feeling of relationship closeness and/or mention the previous decrease in cooperation and possible reasons thereof. The communication content is thus partly quite similar to the external “expert” explanation and advice we give to the participants adding a personal nuance, which could be important.

To end on a somewhat speculative note, it could make a difference whether the information is transmitted from within the group or from outside the group (Mackie et al. 1992). Based on the results of a field experiment on information provision on people’s earnings, Chetty and Saez (2013) for instance conclude that knowledge transfer through peer networks among others could have a larger impact on people’s behavior than simple information provision by experts. Also, the content of the “expert” explanation and advice is purely informative (production oriented) while the leaders can evoke feelings and emotions such as identity, solidarity, or guilt for letting others down and praise the observed cooperation behavior (people oriented), which they do more often when they are not influenced by the expert explanation and advice. Another possibility could be that too much information is not good for changing individuals’ behavior. Also the leader can target the previous cooperation in the own group with the free form communication, while the comprehension/advice text is a general statement. It would be interesting to analyze in future work what kind of communication leaders can use to restore cooperation in organizations.

## Electronic supplementary material

Below is the link to the electronic supplementary material.

Supplementary material 1 (DOCX 701 kb)

## Footnotes

1 See Davis and Holt (1994) and Ledyard (1995) for reviews and Fischbacher and Gächter (2010) for a recent analysis of cooperation decay.

2 Leadership can also be studied experimentally without a leading-by-example structure. See, for example, Brandts et al. (2014).

3 In Boulou-Reshef et al. (2014) leaders move first by sending a message to followers but they cannot make moves in ways that set an example.

4 Assuming rationality, selfish preferences and common knowledge of rationality the equilibrium contribution of leaders and followers in the sequential structure of the game is the same as in the simultaneous game, i.e. zero (individual payoff of 40 ECU). This holds for the stage game as well as for the finitely repeated game, which can be shown by backward induction. The socially optimal solution is just the same as in the finitely repeated simultaneous game: Each group member contributes in each round the entire individual endowment to the public good leading to a total contribution of 160 (individual payoff of 80 ECU).

5 Note that Andreoni (1988) and Croson (1996) studied the effects of a pure restart in a simultaneous voluntary contribution game and the restart was a surprise for participants. Hence, our control treatment is an extension and not a pure replication of previous work. To our knowledge, the restart effect as such has not yet been studied in a sequential form of the game and without it being a surprise.

6 See, e.g., Drexler et al. (2010), Karlan and Valdivia (2011), Bruhn and Zia (2011), Bruhn et al. (2012), Karlan et al. (2012), Bloom et al. (2013). Compared to the advice provided in Chaudhuri et al. (2006), where common knowledge advice coming from a *non-expert* participant from a *previous generation* increases cooperation, our advice has the nature of an *exogenous expert advice*.

7 We formulated the advice in the comprehension/advice treatment in a clear, but comprehensive way to avoid that participants interpreted it as an order.

8 We thought carefully about the information we put in the explanation and advice and let non-economists proofread it for understandability. Also, we gave participants enough time to read the information again after we had read it out aloud and asked if anyone had a question before proceeding.

9 All our treatments involve a repetition at stage 3.

10 Bayer et al. (2013) study a repeated simultaneous public goods game. They include a treatment in which participants do not get initial information about the specifics of the game and its payoff structure and compare behavior in this case with that in a standard information condition. They find that after a few rounds, the rate of decline was much lower with no information so that in their framework confusion does not lead to a faster decline.

11 Note that the informational content and understanding given to participants in the comprehension/advice treatment can be considered to be at least as precise and deep as in the communication treatment.

12 In what follows all pair-wise comparisons are based on two-sided tests.

13 The coefficient estimate of the treatment C dummy (4.4) has a p value of 0.114. The difference in the slope seems to be a random effect since there are no design differences between the treatments in part 1.

14 Also the highly significant coefficient estimate of the part 2-dummy in regression model (4a) in Table 3 confirms an average increase of 11.57 ECU from round 12 to round 13 (part 2—coefficient estimate: 12.37 ECU; marginal round change: −0.80 ECU).

15 For treatment CAC the increase is not significantly higher than for treatment PR, although the average contribution level in round 13 for CAC is as high as for treatment C. The lack of significance can be attributed to the fact that the contribution level in treatment CAC (randomly) remained relatively high in round 12.

16 We run fixed effects regressions (robust standard errors) for each treatment and each part separately regressing the individual contributions on the round variable (see Online Appendix A.6 for the regression results). Except for treatment C in part 3 (p = 0.303), the coefficient estimates for the round variable are significantly negative for all other eleven treatment and part combinations, i.e. also for all treatments in part 2 (p < 0.052).

17 Note also that like in part 1 leaders contribute more then followers.

18 We can also test for the differences of the coefficients in regression (4b). The only significant difference we find is between *(C)*(Part 2)* and *(CA)*(Part 2)* (p = 0.0876, Wald post estimation test).

19 Throughout we have focused on average effects across all groups. To see if this hides differences between types of groups we also do the treatment-effect analysis separately for those groups who experience a decline (and those who do not). We define as groups who experience a decline those with a significant average decline per round in part 1 of at least 0.5 (p value <0.1 in Spearman rank correlation, Kendall rank correlation, OLS of average group contribution on round, and FE regression of individual contribution on round). This is in fact the case if, in the FE regression, the average decline per round in part 1 is at least 0.9 (p value <0.1), which corresponds to the average decline per round in regression models (1a) and (1b) in Table 3. Based on this procedure, we exclude 7 (treatment PR), 4 (treatment CA), 4 (treatment C), and 5 (treatment CAC) groups from the analysis. We repeat the regression analysis in Table 3 for the remaining groups and confirm overall the findings with all group observations underlining the robustness of our results. (We thank one of the reviewers for suggesting this interesting additional analysis).

20 The rise in cooperation from part 1 to part 2 is only significant with communication; both without (p = 0.050, Wilcoxon signed ranks test) and with (p = 0.060, Wilcoxon signed ranks test) comprehension and advice. In both treatments, contributions increase by around 35 %.

21 Note, however, that the increases are 9 to 10 times the one for treatment CA.

22 Testing for differences of the interaction coefficients for part 3 in regression (4b) we find that *(C)*(Part 3)* is significantly larger than *(CA)*(Part 3)* (p = 0.0229, Wald post estimation test). All other tests between the interaction terms in model (4b) are insignificant.

23 That the repeated effect is stronger in treatment C than in treatment CAC is also confirmed in the separate analysis for groups with declining cooperation in part 1. See footnote 19 for the definition of groups with declining cooperation.

24 A Wald post estimation test shows that the coefficient estimate of the interaction term *(C)*(Part 3)* is larger than the coefficient estimate of the interaction term *(C)*(Part 2)* though insignificantly, model (4b) of Table 3 (p = 0.1254). For the other two intervention treatments CA and CAC, there are clearly no differences between the coefficient estimates of the corresponding iteraction terms (p > 0.774, Wald post estimation test).

25 One of the co-authors did the coding of the text messages as objectively as possible.

26 Labor notions refers to the leader using the words: worker, manager, firms.

27 Due to technical problems, the message of one leader was not saved.
