# Supplementary material for: Not just like starting over - Leadership and revivification of cooperation in groups
Source: Exp Econ. 2015 Sep 25;19(4):792–818. doi: 10.1007/s10683-015-9468-6 (PMC5153666; doi:10.1007/s10683-015-9468-6)
Supplement: Supplementary file 1 — Supplementary material 1 (DOCX 701 kb) [file 10683_2015_9468_MOESM1_ESM.docx]

**Appendix**

**Instructions**

***A.1. Instructions at the beginning of the experiment***

**General information**

Thank you for coming to the experiment. You will receive 5 Euro for the participation in the experiment. You will be assigned to a group and depending on your and your group members’ decisions you can earn additional money during the experiment. It is important that you do not talk to any of the other participants until the experiment is over. You can ask questions at any time. If you have a question, please raise your hand and one of us will come to your place to answer.

**Role and group matching**

You will be randomly assigned to one of two roles: (1) director or (2) employee. This role will be the same throughout the entire experiment.

Participants will be randomly split in groups with 4 members, each composed by 1 director and 3 employees. At no time during the experiment you will know whom you are matched with and your decisions will be anonymous.

**Task and stages of each of the 36 rounds**

There will be 36 separate rounds. In each round, each group works on a joint project whose payoff will depend on the hours dedicated by all group members. In each round, every participant has an endowment of 40 hours and decides how many of the 40 hours to dedicate to the project. The remaining hours will be automatically dedicated to a private activity.

Each round is independent from the others and develops in the following way:

Stage 1:

Directors: The director of each group decides how many of the 40 hours to dedicate to the project. The rest will be automatically dedicated to the private activity. There will be a simulation area on the lower part of the screen where directors can calculate earnings choosing different hours dedicated to the project by themselves and by the other group members on average (see “Decision screen director”). The calculations are absolutely private. In the upper part of the screen, directors enter the hours that they want to dedicate to the project in the corresponding round.

Employees: The employees do not have anything to do in this stage and wait until the director of their group have taken a decision.

Stage 2:

Directors: The directors do not have anything to do in this stage and wait until the employees of their group have taken a decision.

Employees: The employees of each group are informed about the hours that the director of their group decided to dedicate to the project and decide how many of their own 40 hours to dedicate to the project. The rest will be automatically dedicated to the private activity. There will be a simulation area on the lower part of the screen where employees can calculate earnings choosing different hours dedicated to the project by themselves and by the other group members on average (see “Decision screen employee”). The calculations are absolutely private. In the upper part of the screen, employees enter the hours that they want to dedicate to the project in the corresponding round.

Stage 3:

Directors and employees: All participants are informed about the average hours dedicated to the project by the other group members, the sum of hours dedicated to the project by all group members and about their own earnings. Summaries of previous rounds will also be listed.

After stage 3, a new round starts which develops in the same way.

**Additional information**

The experiment is split in 3 parts and each part consists of 12 rounds. The specific instructions for each part will be shown on the screen before the corresponding part starts.

**Payoff**

Your earnings in Experimental Currency Units (ECU) for each round are given by the following function, which is the same for directors and employees:

$$Earnings_{Round}=\underbrace{\left(40-Hours_{\Pr oject}\right)}_{\text{Earnings from private activity}}+\underbrace{0.5\cdot\sum_{Group} Hours_{\Pr oject}}_{\text{Earnings from joint project}}$$

The earnings in ECU are composed by the earnings from the *hours* dedicated to the *private activity* by that person and the earnings from the *sum of hours* dedicated by *all group members* to the *joint project*. That means that each hour that you decide to dedicate to the project gives *each* of the group members (i.e. you and all other group members) an earning of 0.5 ECU. Analogously, each hour that another group member decides to dedicate to the project gives *each* of the group members (i.e. you and all other group members) an earning of 0.5 ECU. Each hour that you decide *not* to dedicate to the project (i.e. to dedicate to the private activity) gives you and only you an earning of 1 ECU.

150 ECU are worth 1.00 Euro. At the end of the session you will receive 5 Euro plus the sum of what you will have earned in all 36 rounds of the experiment. After the experiment finishes we will pay you the earnings in private.

**Example and test question**

So that everyone understands how decisions translate into earnings we provide an example and a test question. (The number of hours used for the example and test are simply for illustrative purposes. In the experiment the allocations will depend on the actual decisions of the participants.)

Example: Suppose that you decide to dedicate 31 hours to the project and the other group members decide to dedicate on average 33 hours to the project in one of the 36 rounds.

The sum of hours dedicated to the project by all group members is:

31 + 3*33 = 31 + 99 = 130 (hours)

Your earnings in that round are:

(40 – 31) + 0.5*130 = 9 + 65 = 74 (ECU)

Test: Suppose that you decide to dedicate 28 hours to the project and the other group members decide to dedicate on average 24 hours to the project in another of the 36 rounds.

The sum of hours dedicated to the project by all group members is:

_________________________________________________________________

Your earnings in that round are:

_________________________________________________________________

**Graphical representation of the chronological order of the experiment**

1 round: 3 stages

Stage 1

Stage 2

Stage 3

Part 2: 12 rounds

Part 1: 12 rounds

Part 3: 12 rounds

Instruction for each part

Payment and questionnaire

Experiment: 36 rounds

Start experiment

End experiment

**Screenshots**

Decision screen director


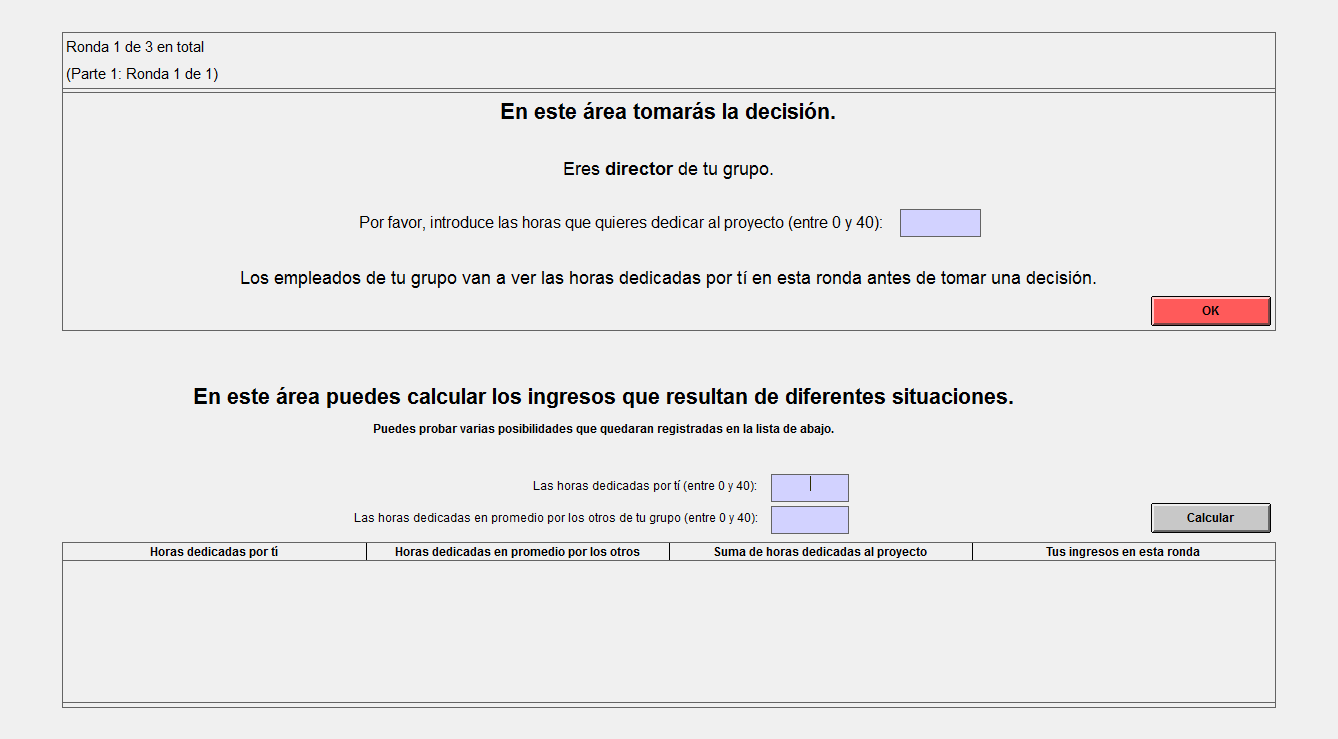


Decision screen employee

(The number of hours used for the example and test are simply for illustrative purposes. In the experiment the allocations will depend on the actual decisions of the participants.)


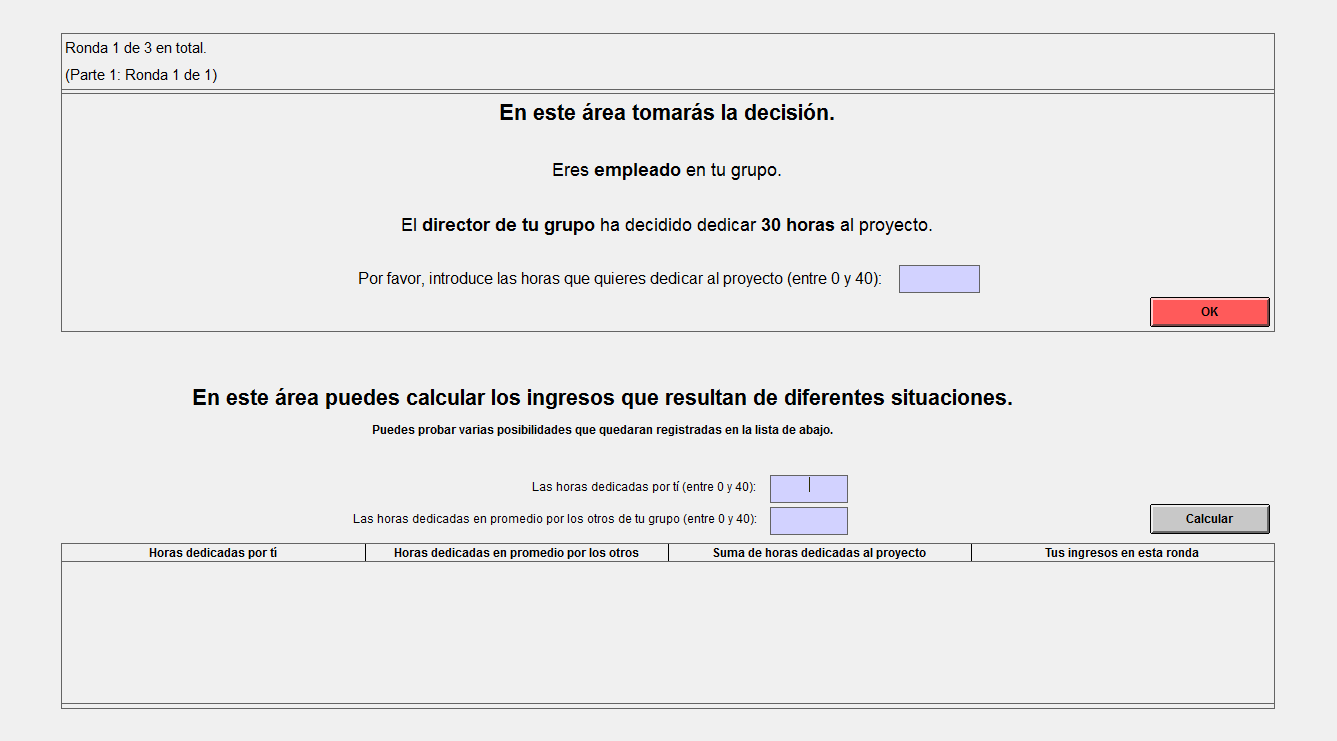


***A.2. Instructions at the beginning of part 1 (all four treatments R, CA, C, and CAC)***

*
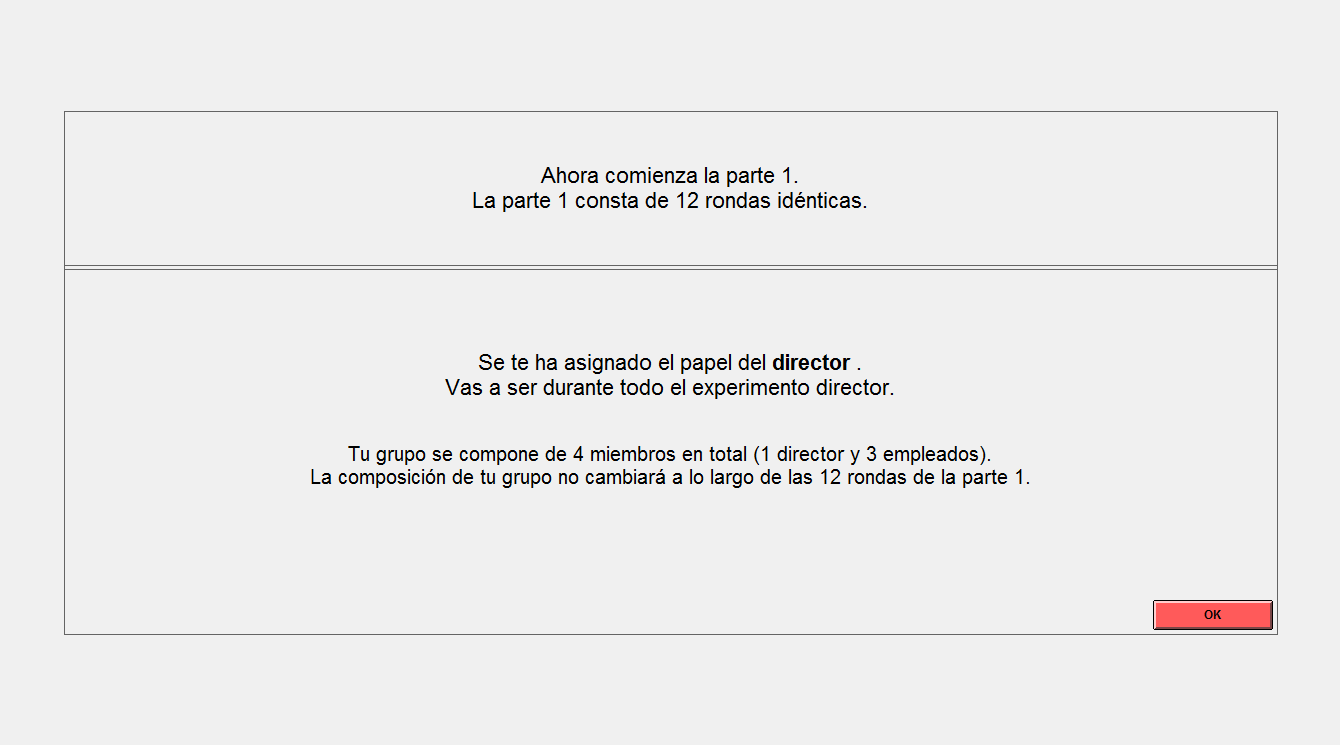
*

*
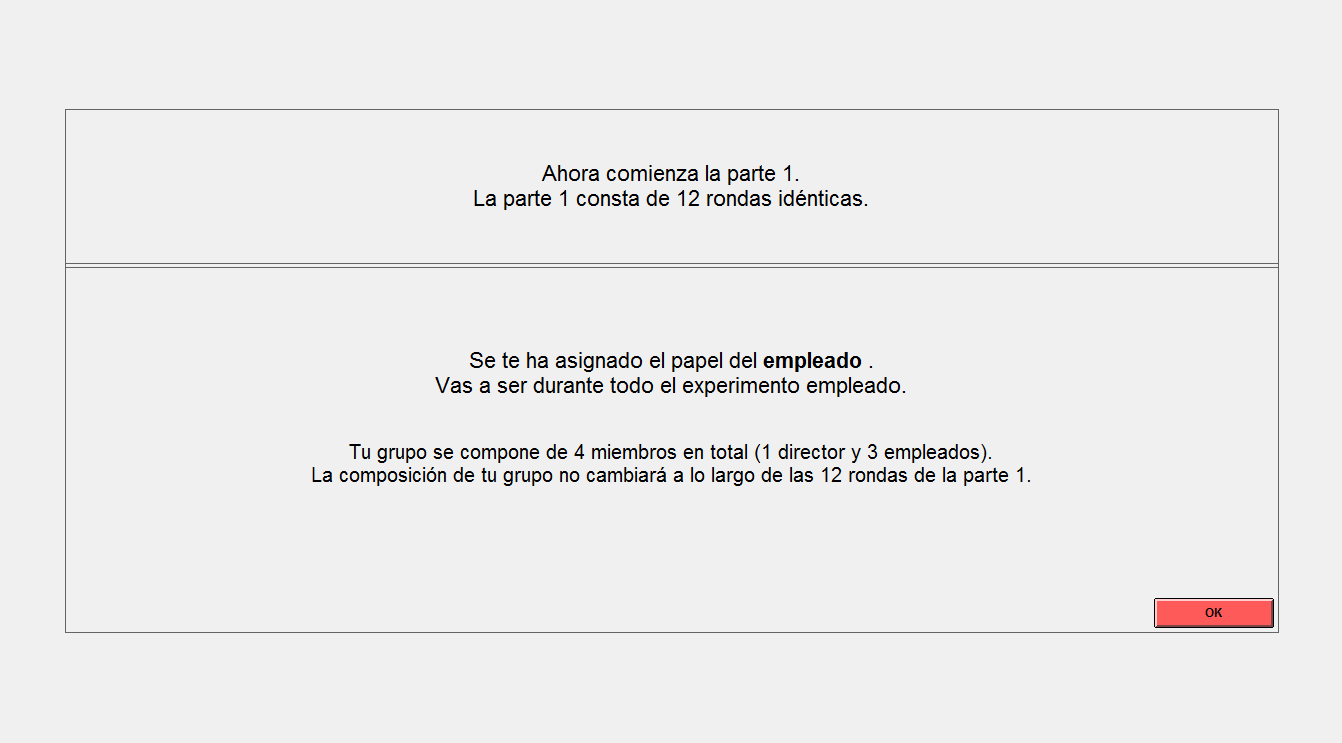
*

***A.3. Instructions at the beginning of part 2 (treatments R, CA, C, and CAC)***

**
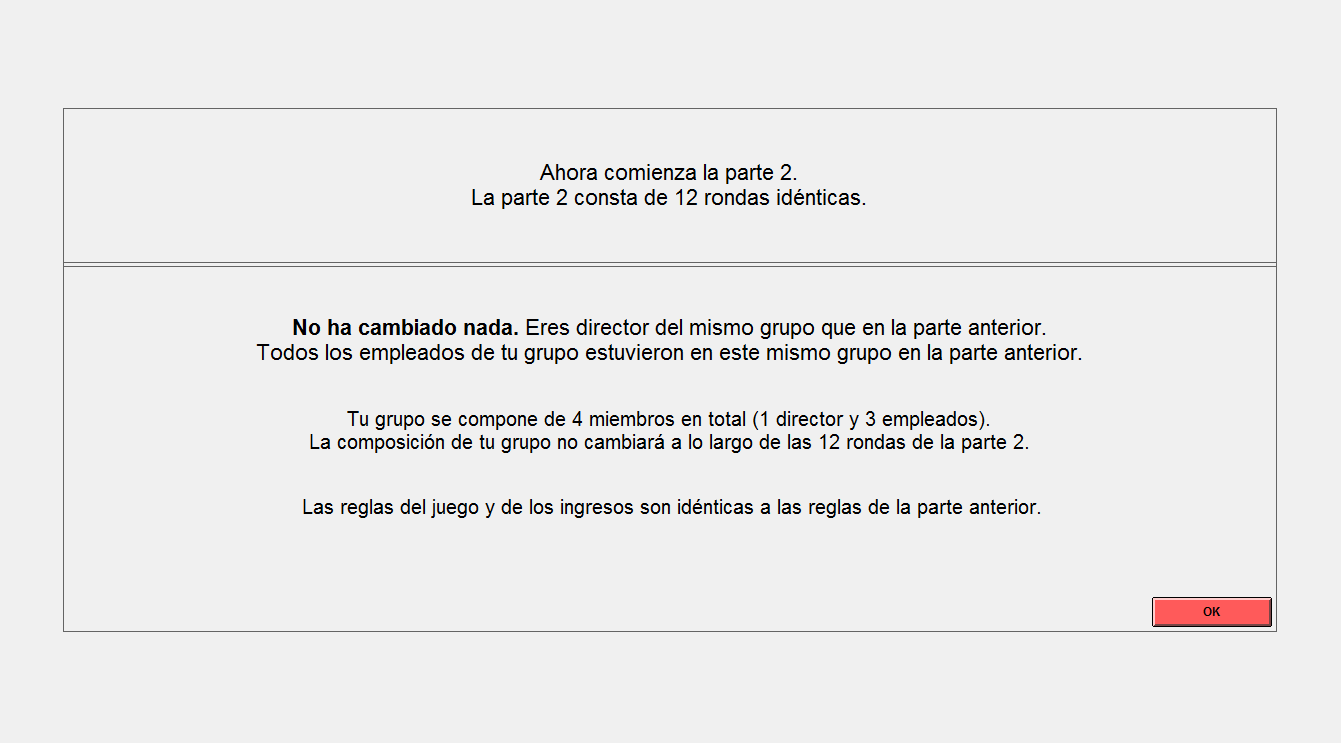
**

**
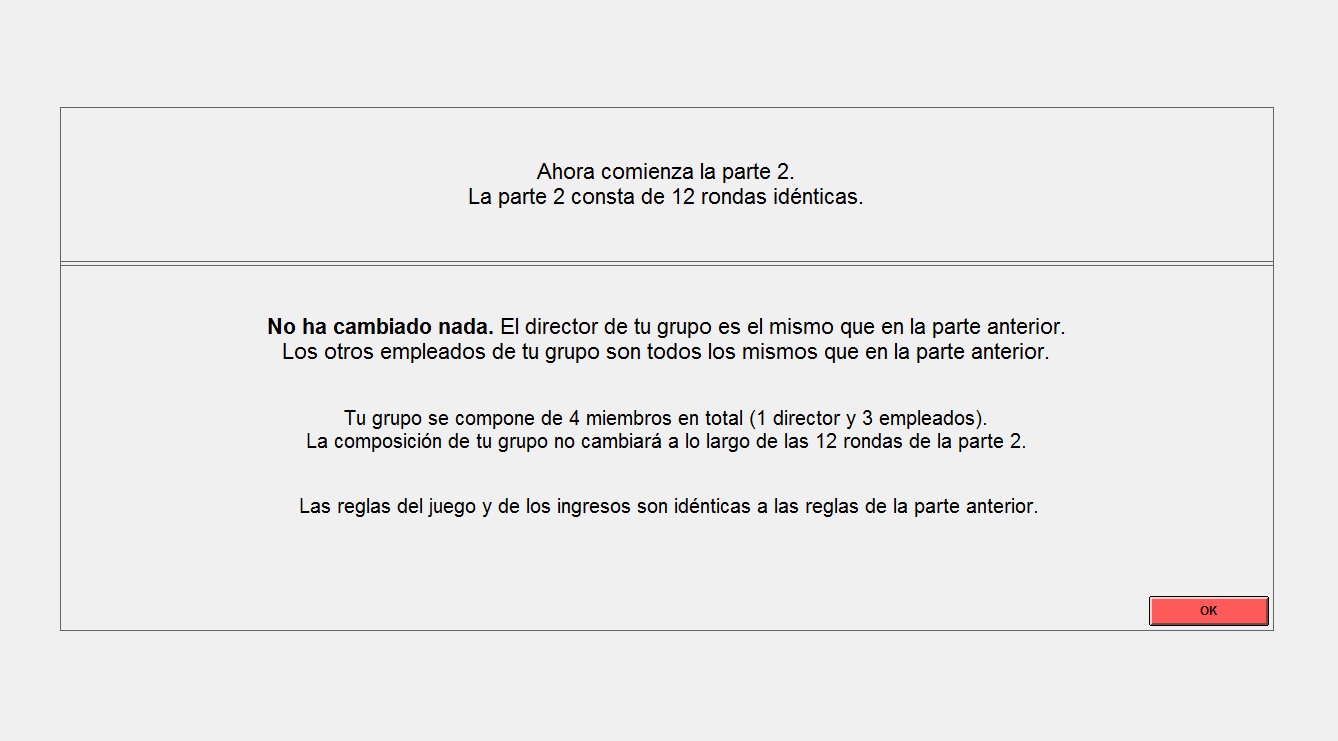
**

***A.4. Additional instructions at the beginning of part 2 and part 3 (treatment CA and CAC)***

Text at the beginning of part 2


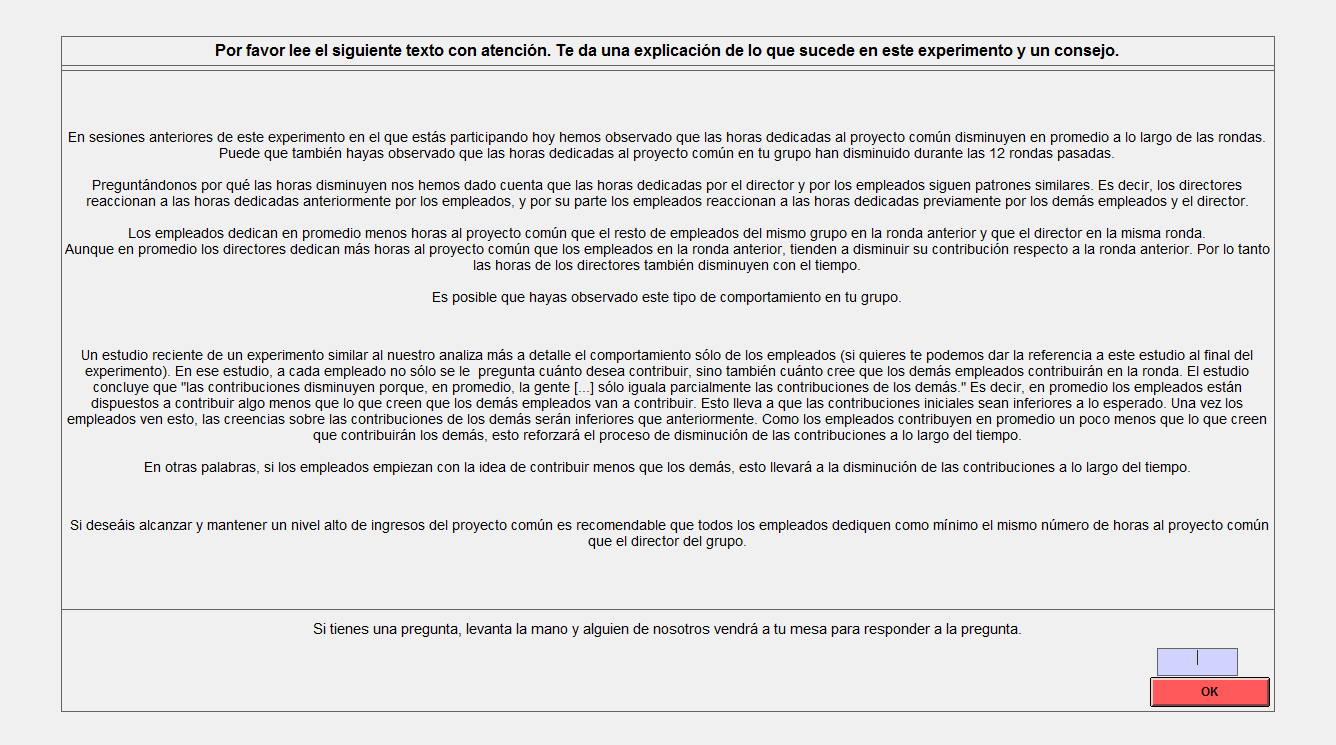


Please read the following text carefully. It gives you some explanation about the game that you are playing in this experiment and some advice.

We observed in previous sessions of this experiment in which you are participating today that the hours dedicated to the common project decrease on average over rounds in this part. You also might have observed that the hours dedicated to the common project in your group decreased over the previous 12 rounds.

We were wondering why contributions decrease and realized that the director’s and the workers’ hours dedicated to the common project follow similar patterns. That means that directors react to the workers’ previous contributions and workers on their turn react to the other workers’ and the director’s previous contributions.

Workers contribute on average fewer hours to the common project than the other workers of the same group in the previous round and less hours than the director in the same round.

Even though the directors dedicate on average more hours to the common project than the workers in the previous round, they also tend to decrease their contributions compared to the previous round. Therefore, the hours of the directors also decrease over time.

You might have observed this contribution behavior in your group.

A recent study of an experiment similar to ours analyzes more in detail the behavior of the workers only in the experiment (if you want, we can provide you with the reference of the study at the end of the experiment). In that study, the workers are not only asked about how much to contribute, but also about what they believe the other workers will contribute. The study concludes, that "contributions decline because, on average, people […] match others’ contributions only partly.” That means that, on average, the workers are willing to contribute slightly less than what they believe the other workers will contribute. This leads to contributions being initially lower than expected. Once workers see this the beliefs about the others’ contributions will be lower than before. Since the workers contribute on average slightly less than what they believe that the others contribute, this reinforces the process by which average contributions decrease over rounds.

In other words, if the workers start with the idea of undercutting others then others will follow and the contributions to the common project will fall over time.

If you wish to reach and maintain a high earnings level from the common project it is recommendable that all workers dedicate at least the same number of hours to the common project as the director of the group does.

If you have a question, raise your hand and someone of us will come to your place to answer the question.

Text at the beginning of part 3


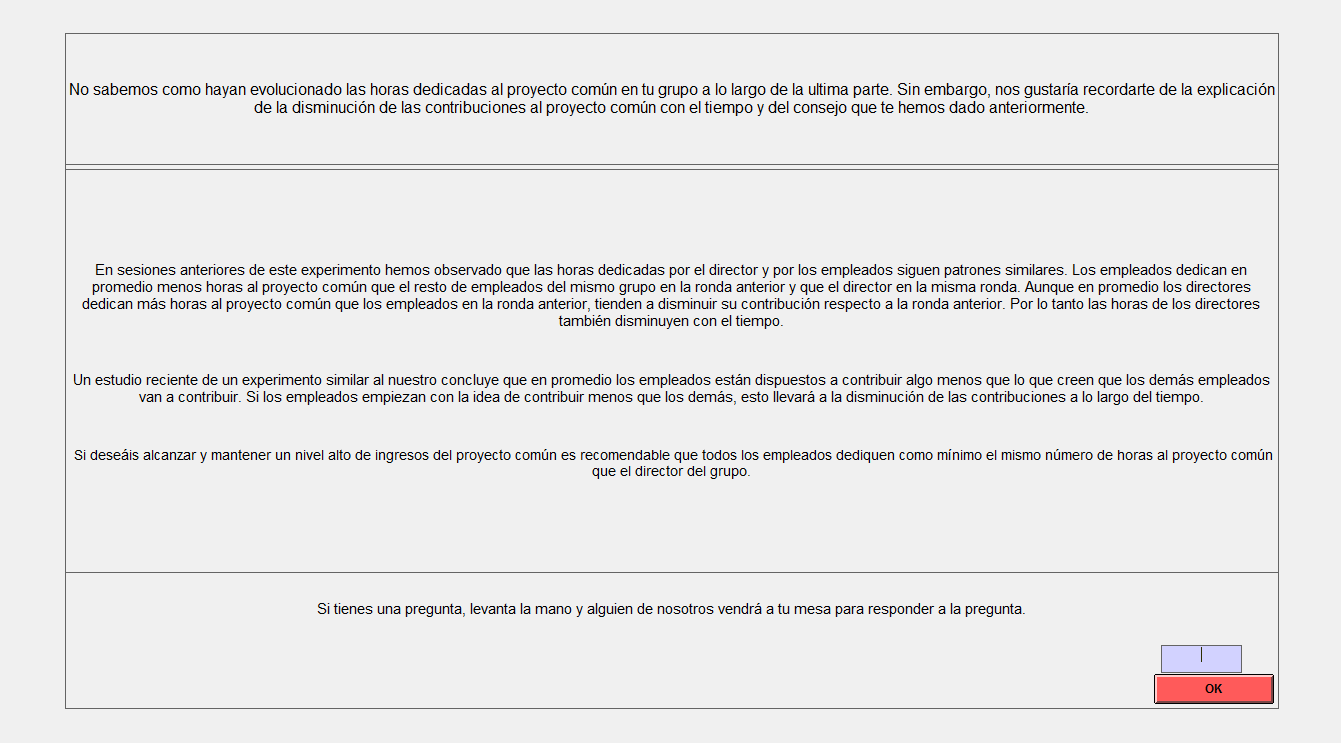


We do not know how hours dedicated to the common project evolved in your group over the previous part. However, we would like to remind you of the explanation for the decline of contributions to the common project over time and the advice that we gave you previously:

We observed in previous sessions of this experiment that the director’s and the workers’ hours dedicated to the common project follow similar patterns. Workers contribute on average fewer hours to the common project than the other workers of the same group in the previous round and less hours than the director in the same round. Even though the directors dedicate on average more hours to the common project than the workers in the previous round, they also tend to decrease their contributions compared to the previous round. Therefore, the hours of the directors also decrease over time.

A recent study of an experiment similar to ours concludes that, on average, workers are willing to contribute slightly less than what they believe the other workers will contribute. If the workers start with the idea of undercutting others, this will lead to the decrease of contributions over time.

If you wish to reach and maintain a high earnings level from the common project it is recommendable that all workers dedicate at least the same number of hours to the common project as the director of the group does.

If you have a question, raise your hand and someone of us will come to your table to answer the question.

***A.5. Additional instructions at the beginning of part 2 and part 3 (treatment C and CAC after having received the comprehension and advice text)***


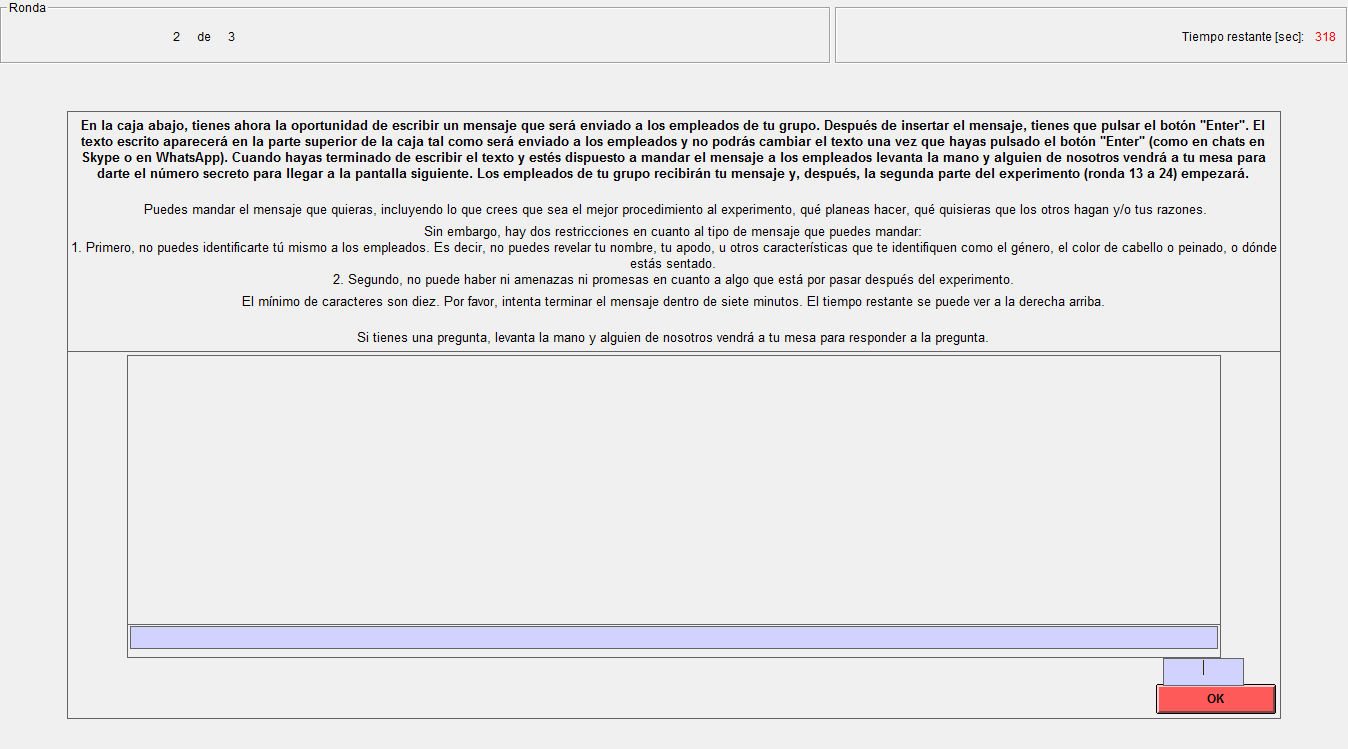


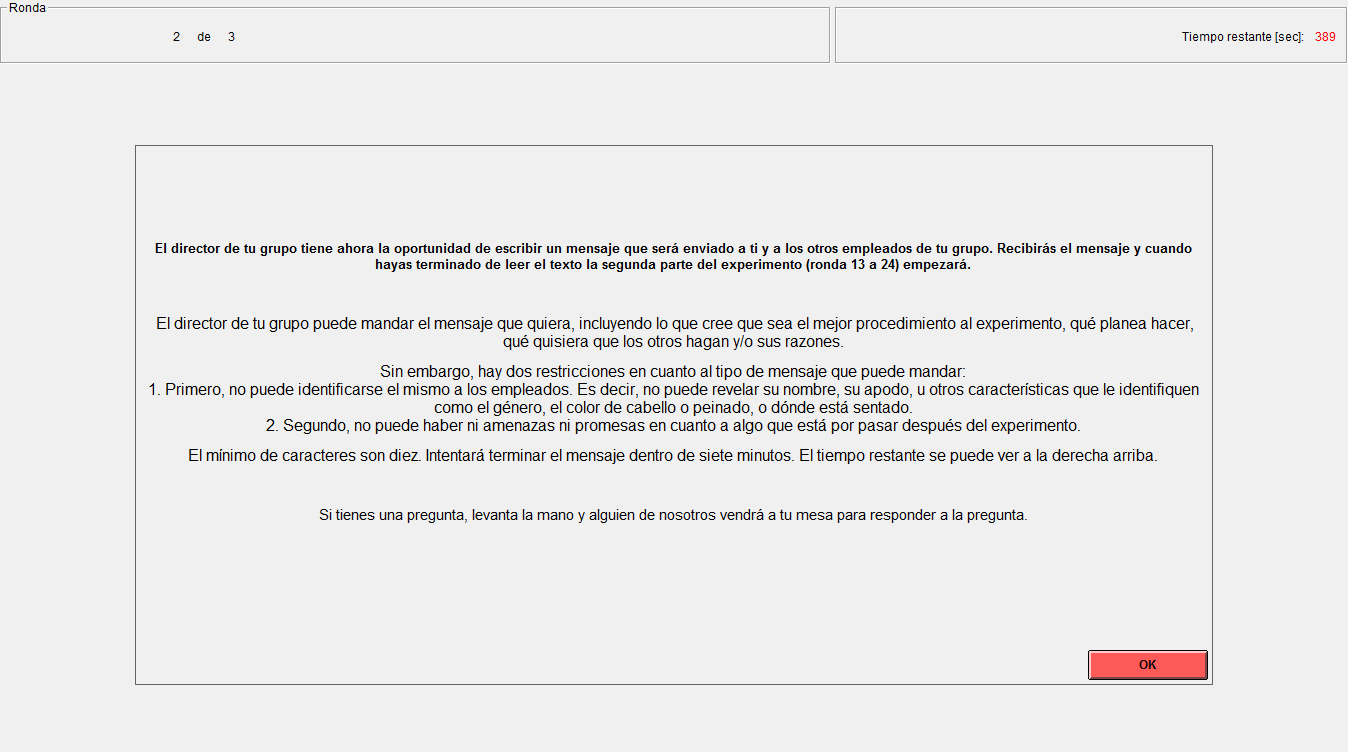


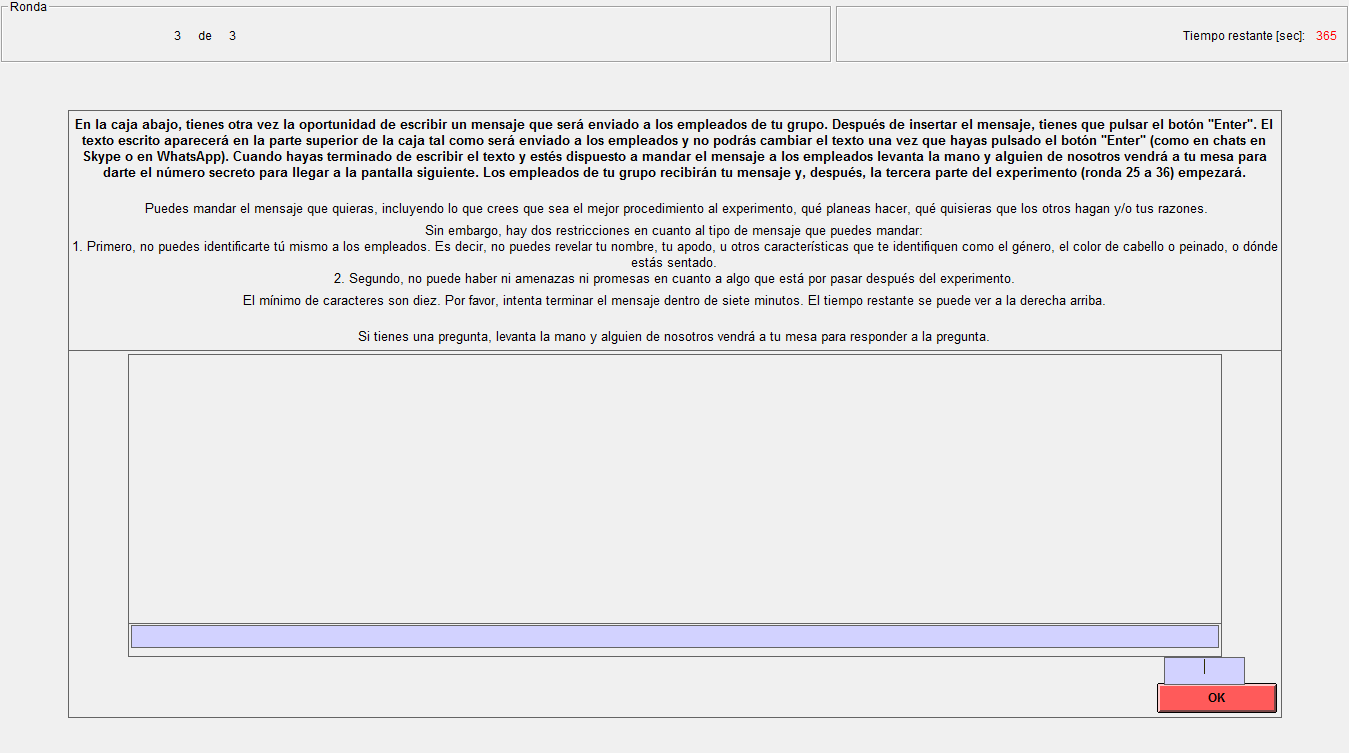

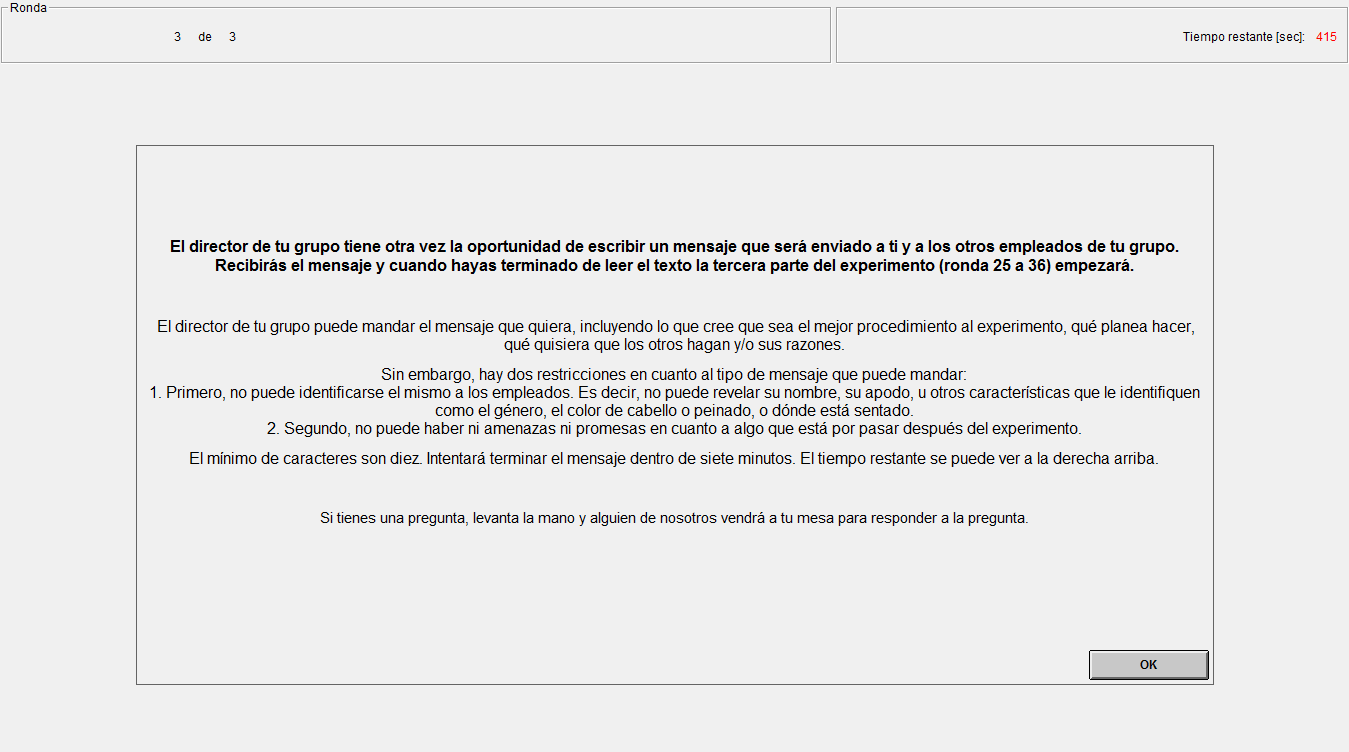


In the box on their screen, the directors have *now/again* the opportunity to write a message, which will be sent to the employees of their group. After entering the message, you - the director - need to press the Enter key. The written text will appear in the upper part of the box the way it will be sent to the employees and you won't be able to change the entered text once you press the Enter key (just like in chats in Skype or WhatsApp). When you have finished writing the text and are ready to send the message to the employees you may raise your hand and one of us will come to your table to give you the code to get to the next screen.^[[1]](#footnote-1)^ The employees will receive the message of the director of their group and, after that, the *second/third* part of the experiment (rounds *13/25* through *24/36*) will start.

You – the director - are free to send the message you like, including what you think is the best approach to the experiment, what you plan to do, and/or what you would like the others to do and/or why. However, there are two restrictions on the kind of messages that you can send:

1. First, you are not allowed to identify yourself to the others. Thus, you cannot reveal your real name, nicknames, or any other identifying feature such as gender, hair, or where you are seated.

2. Second, there must be neither threats nor promises pertaining to anything that is to occur after the experiment.

The minimum entry of characters is 10. Please, try to finish your message within seven minutes. The remaining time in seconds is shown on the upper right corner of the screen.

If you have a question, raise your hand and someone of us will come to your table to answer the question.

**Decline of cooperation**

***A.6. Fixed-effects regression of individual contribution on round variable***

|  |  |  |  |  |
| --- | --- | --- | --- | --- |
|  | Treatment PR | Treatment CA | Treatment C | Treatment CAC |
|  |  |  |  |  |
| Part 1 | -0.59 | -1.11 | -1.22 | -0.66 |
|  | (0.163) | (0.164) | (0.143) | (0.223) |
|  | *0.001* | *0.000* | *0.000* | *0.005* |
|  |  |  |  |  |
| Part 2 | -0.75 | -1.15 | -1.11 | -0.38 |
|  | (0.171) | (0.188) | (0.260) | (0.188) |
|  | *0.000* | *0.000* | *0.000* | *0.051* |
|  |  |  |  |  |
| Part 3 | -0.67 | -1.32 | **-0.15** | -0.52 |
|  | (0.179) | (0.182) | **(0.146)** | (0.253) |
|  | *0.000* | *0.000* | ***0.302*** | *0.047* |
|  |  |  |  |  |
| Fixed-effects regression (robust standard errors) of individual contribution on round variable. | | | | |
| Reported values are the coefficient estimate for the round variable, the standard error (in parenthesis) and the corresponding p-value (cursive). | | | | |
| All individual observations for each treatment and part combination are used. | | | | |

**Coding of communication**

***A.7. Description of coding categories***

The first five coding categories capture the content of the comprehension/advice message in treatment CA (and CAC). The intention is to see whether leaders mention an observed decline in previous contributions, whether they observed followers undercutting in general, whether they mention one or more possible explanation(s) such as selfishness and consequences of such an undercutting behavior, i.e. others may follow the example. Finally, we code a request for conformity, i.e. the leader’s emphasis on the need that all group members conform to the leader’s contribution.

The next six categories involve payoff-related arguments. In particular, they include the leader’s suggestion (point or interval) of how much to contribute to the project; the suggestion, implicit or explicit, must be unambiguous. We code whether an implicit or explicit suggestion is that everybody in the group (including the leader) contributes the whole endowment. Furthermore, coding categories enclose whether the leader makes explicit payoff calculations associated with the proposal, whether he argues explicitly that the suggested amount maximizes the group payoff, or conjectures that participants are interested in maximizing the group payoff, as well as whether the leader mentions explicitly that the followers benefit from following his suggestion. Finally, the last category in the payoff-related group captures whether the leader announces punishment in reaction to followers defecting the suggested contribution level. The four mentioned punishment strategies were tit-for-tat, two-tit-for-tat, grim trigger, and random/reducing contribution if a follower defected.

The third group of coding categories encompasses social preferences, emotional expression, and own contribution behavior. With fairness, we refer to an explicit or implicit reference to fairness or just behavior, which also includes an explicit rejection of some group member contributing less than the others. Team spirit refers to a statement promoting the willingness to cooperate as part of a team or emphasizing the importance of cooperation in the group. Closely related is the notification of low contributors, implicit or explicit, of those who contributed less than suggested or who started decreasing their contributions. Here, leaders point to a particular group member undercutting the others’ contributions (e.g. a statement like “there must be a group member undercutting the others’ contributions”). Group leaders may conjecture this if for instance the average followers’ contributions are 80 ECU (= 40 ECU + 40 ECU + 0 ECU as one option). Note that in the category “Observation of followers undercutting,” the leader refers to the followers as a whole. We furthermore code whether the leader praises or complains about observed contributions. The mood of the communication is (mostly) independent from the leader’s praise or complaint and gives an overall impression of bad, neutral, or positive vibes, which includes the use of “smileys,” or other forms of creating a good or bad atmosphere. Furthermore, we code whether the leader leaves the contribution decision explicitly to the followers, promises to contribute some specific amount, or expresses the willingness to contribute more than the followers do.

The last group includes two coding categories. We code whether the leader uses the labor notion from the instructions, e.g. “director,” “workers,” or “firm,” and whether the communication content is to some extent strange, wrong or does not make sense. The number of analyzed text messages in round 13 in treatment CAC is 11 (due to technical problems, the message of one leader was not saved). In all other cases 12 text messages were analyzed, respectively. We also coded whether the form of the text message is informal, neutral or formal (not reported in the table), but do not find significant differences.

1. A reviewer remarks that by rasing their hands leaders more or less had to identify themselves and that this could have affected behavior. Even though it may not be known which leader belongs to which group, this may have an effect. However, most often participants raised their hand in a rather unconspicouos way. We did not get the impression that subjects paid any attention to this, so that we are quite sure it had no effect. We did this to avoid unnecessary delays in the sessions which were already quite long. [↑](#footnote-ref-1)
